# LINC01016 promotes the malignant phenotype of endometrial cancer cells by regulating the miR-302a-3p/miR-3130-3p/NFYA/SATB1 axis

**DOI:** 10.1038/s41419-018-0291-9

**Published:** 2018-02-21

**Authors:** Xin Pan, Da Li, Jianing Huo, Fanfei Kong, Hui Yang, Xiaoxin Ma

**Affiliations:** Department of Obstetrics and Gynecology, Key Laboratory of Maternal-Fetal Medicine of Liaoning Province, Key Laboratory of Obstetrics and Gynecology of Higher Education of Liaoning Province, Shengjing Hospital of China Medical University, 39 Huaxiang Road, Shenyang, 110021 People’s Republic of China

## Abstract

Long noncoding RNAs (lncRNAs) have been implicated in tumorigenesis and cancer progression and are tightly associated with the phenotypes of numerous cancers. However, the functional roles underlying these effects are unknown. The expression levels of LINC01016, miR-302a-3p, miR-3130-3p, NFYA, and SATB1 were evaluated by quantitative real-time polymerase chain reaction (qRT-PCR) in 33 endometrial cancer tissues and 20 normal tissues. Bioinformatics analyses, luciferase reporter analyses, chromatin immunoprecipitation (ChIP) assays, and qRT-PCR assays were performed to verify potential binding sites. The qRT-PCR and western blot were used to identify the regulatory mechanisms of LINC01016 in cell biological behavior, which were also examined by cell counting kit -8 (CCK-8), 5-ethynyl-2′-deoxyuridine (EdU) assays, flow cytometry, wound healing assays, and transwell assays. LINC01016 was substantially upregulated in endometrial cancer tissues, and LINC01016 silencing abolished the malignant behavior of endometrial cancer cells. LINC01016 positively rescued the downstream gene nuclear factor YA (NFYA) by competitively “sponging” miR-302a-3p and miR-3130-3p. In turn, these two miRNAs could inhibit LINC01016 transcription, thus forming two reciprocal repression cycles, which influenced the biological behavior of endometrial cancer cells. MiR-302a-3p and miR-3130-3p could specifically bind with the 3′-UTR regions of NFYA, and NFYA could upregulate the expression of special AT-rich sequence-binding protein 1 (SATB1) as a transcriptional factor. This study was the first to show that the LINC01016–miR-302a-3p/miR-3130-3p/NFYA/SATB1 axis played a crucial role in the occurrence of endometrial cancer. These findings may provide relevant insights into the diagnosis and therapy of endometrial cancer.

## Introduction

Endometrial cancer is the fifth most common gynecological malignancy in women worldwide. Regardless of rigorous screening and extensive prophylaxis^1,2^, the annual incidence of endometrial cancer continues to increase in many countries^3^. Endometrial cancer is a complicated disease associated with diverse disorders that are involved in its etiology, pathology, and clinical manifestation^4^. For example, female hormonal factors, body mass index, diabetes mellitus, genetic inheritance, and diet quality have all been linked to endometrial cancer development^5–9^. Endometrial cancer is usually diagnosed at an early stage because of abnormal vaginal bleeding. Although a hysterectomy together with radiotherapy and a lymphadenectomy is associated with a statistically significant reduction of non-cancer mortality in stage I and II endometrial cancers, the prognosis and survival rate of advanced, metastatic endometrial cancer remain points of concern^10^. Therefore, the underlying genetic alterations that initiate endometrial cancer need to be elucidated to understand the potential mechanisms of endometrial cancer development. This knowledge is crucial for the establishment of therapeutic targets.

Long noncoding RNAs (lncRNAs) are a class of noncoding RNAs with lengths exceeding 200 nucleotides (nt). LncRNAs contribute to transcriptional and post-transcriptional functions^11^ and can broadly be classified as signaling molecules, decoy molecules, guide molecules, or scaffold molecules^12^. Abundant evidence has confirmed that lncRNAs are involved in multiple tumorigenic and oncogenic processes^13^. As we all know, endometrial cancer is an estrogen-associated disease, and mounting studies have revealed a relationship between endometrial cancer and estrogen or estrogen receptor (ER) ^14^. It was reported that LINC01016 was highly expressed in breast cancer and was demonstrated to be a direct transcriptional target of ERα. LINC01016 also showed prognostic significance in relation to breast cancer survival^15^. These findings support the possibility that LINC01016 could be a relevant biomarker in ERα-positive tumors, including those of endometrial cancer.

Unlike lncRNAs, microRNAs (miRNAs/miRs) are relatively conserved, with sequence lengths of 18–22 nt. The miRNAs serve as negative gene regulators by binding the 3’-untranslated region (UTR) of target mRNAs and promoting RNA degradation in mammals^16^. Each miRNA may control various biological processes and, similarly, each biological process may contain numerous miRNAs. According to the concept of competing endogenous RNAs (ceRNAs), RNAs can interact with each other by competing for shared miRNAs, indicating another method of post-transcriptional regulation^17^. Through crosstalk with diverse downstream targets, miR-302a-3p repressed initiation and development of cancer cells, such as breast and prostate cancer cells^18,19^. Nevertheless, the underlying mechanistic basis for the function of miR-302a-3p is not fully understood. To date, no research has been performed on miR-3130-3p.

Nuclear factor YA (NFYA) is one of the three subunits of a ubiquitous protein and is a nuclear transcriptional factor that is highly conserved from yeast to mammals^20^. NFYA was found to show various functions in tumor development^21,22^. For example, NFYA promoted the proliferation of ovarian cancer cells by inducing expression of EZH2^23^. NFYA-short, one of the alternatively spliced isoforms of NFYA, was found to have high transactivation ability in endometrial adenocarcinoma. In cooperation with Oct-1, NFYA acted on the octamer binding motif of aldehyde dehydrogenase (ALDH) as a CCAAT-recognizing transcriptional factor^24^. Moreover, NFYA played a part in the gene regulation of bovine endometrium treated with steroid hormones^25^. We predicted NFYA to be a target of miR-302a-3p and miR-3130-3p by TargetScan. Nevertheless, the role of NFYA in endometrial cancer is still ambiguous.

SATB1 is a nuclear matrix attachment region-binding protein that recently emerged as a new regulator of oncogenic pathways^26^. SATB1 was demonstrated to be involved in a variety of cancers, and its transcript level was obviously upregulated during endometrial carcinogenesis^27^. We predicted that NFYA could act as a transcriptional factor of SATB1 and the promotor of SATB1 would contain a putative CCAAT sequence to which NFYA could bind.

Here, we found that LINC01016 promoted oncogenic processes by targeting miR-302a-3p and miR-3130-3p and inducing the overexpression of NFYA, thus affecting SATB1 expression in endometrial cancer cells. LINC01016 regulated miR-302a-3p and miR-3130-3p through the formation of two negative feedback loops. These findings may provide deeper insights into endometrial cancer therapy.

## Results

### LINC01016 was upregulated in fresh clinical tissues and acted as an oncogene in endometrial cancer cells

**Fig. 1:**
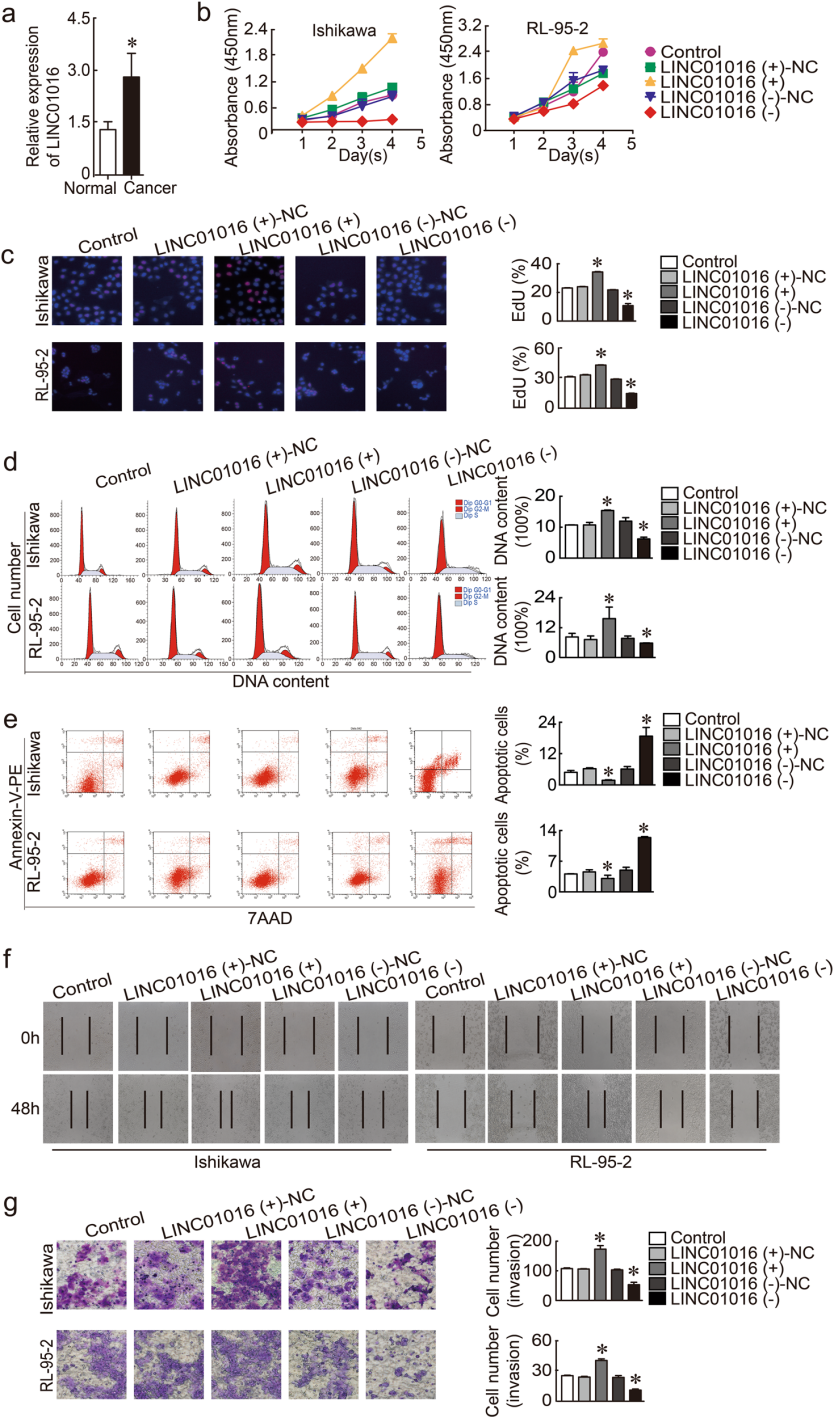
LINC01016 was overexpressed in endometrial cancer tissues and functioned as a pro-oncogene in human Ishikawa and RL-95-2 cell lines . **a** LINC01016 was overexpressed in endometrial cancer tissues (33 cases in the cancer group and 20 cases in the normal unaffected group). **b**, **c** Effect of LINC01016 on the proliferation of Ishikawa and RL-95-2 cell lines, as demonstrated by CCK-8 and EdU assays. **d** Representative flow cytometry images are shown with the cell cycle phases of Ishikawa and RL-95-2 cell lines and the transfection status of LINC01016 is indicated. **e** Representative flow cytometry images of apoptotic Ishikawa and RL-95-2 cell lines with indicated transfection status of LINC01016. **f** Wound healing assay was performed to investigate cell migration capacity. **g** Transwell assays were performed to investigate the quantities of invading cells in response to altered expression of LINC01016. Representative images and accompanying statistical plots are shown. Data are presented as the means ± SD from three independent experiments. *P < 0.05

LINC01016 was more highly expressed in endometrial cancer tissues than in unaffected tissues detected by quantitative real-time polymerase chain reaction (qRT-PCR; Fig. 1a) and in situ hybridization (ISH) (Supplementary Figure S1a). To determine the LINC01016 phenotype, endometrial cancer cell lines were transfected with LV-LINC01016 (+), LV-LINC01016 (−), and the respective scrambled negative control. We designed three lentiviral constructs that knocked down LINC01016 and confirmed transfection efficiency by qRT-PCR. We found that one construct (PSC50860-22) decreased the expression of LINC01016 by 33.1% (Supplementary Figure S2a); therefore, we used this construct in subsequent knockdown experiments. In cell counting kit -8 (CCK-8) assays, the proliferation rate was increased in the LINC01016 (+) group but was decreased in the LINC01016 (−) group (Fig. 1b). Findings of the 5-ethynyl-2′-deoxyuridine (EdU) assay were consistent with the CCK-8 assay (Fig. 1c). We then investigated the role of LINC01016 in cell cycle regulation and apoptosis. We confirmed that ectopic expression of LINC01016 increased the proportion of endometrial cancer cells in G2–M phase. In contrast, LINC01016 knockdown decreased the number of cells in G2–M phase (Fig. 1d). Early apoptotic cells were annexin V–phycoerythrin (PE) (+) 7-aminoactinomycin D (7-AAD) (−) (upper left quadrant) and late apoptotic cells were annexin V–PE (+) 7-AAD (+) (upper right quadrant). The proportion of total apoptotic cells (early and late) significantly decreased as the expression of LINC01016 increased (Fig. 1e). The wound healing assay showed that low expression of LINC01016 impeded migration in Ishikawa and RL-95-2 cell monolayers; LINC01016 overexpression had the opposite effect (Fig. 1f). The results from the transwell assay demonstrated that the number of invading cells in the LINC01016 (+) group was significantly greater than that of the NC group. In comparison, cell invasion in the LINC01016 (−) group was decreased relative to the NC group (Fig. 1g). Therefore, LINC01016 promoted the malignant behavior of endometrial cancer cells.

### miR-302a-3p and miR-3130-3p were weakly expressed in endometrial cancer tissues and functioned as tumor suppressors

**Fig. 2:**
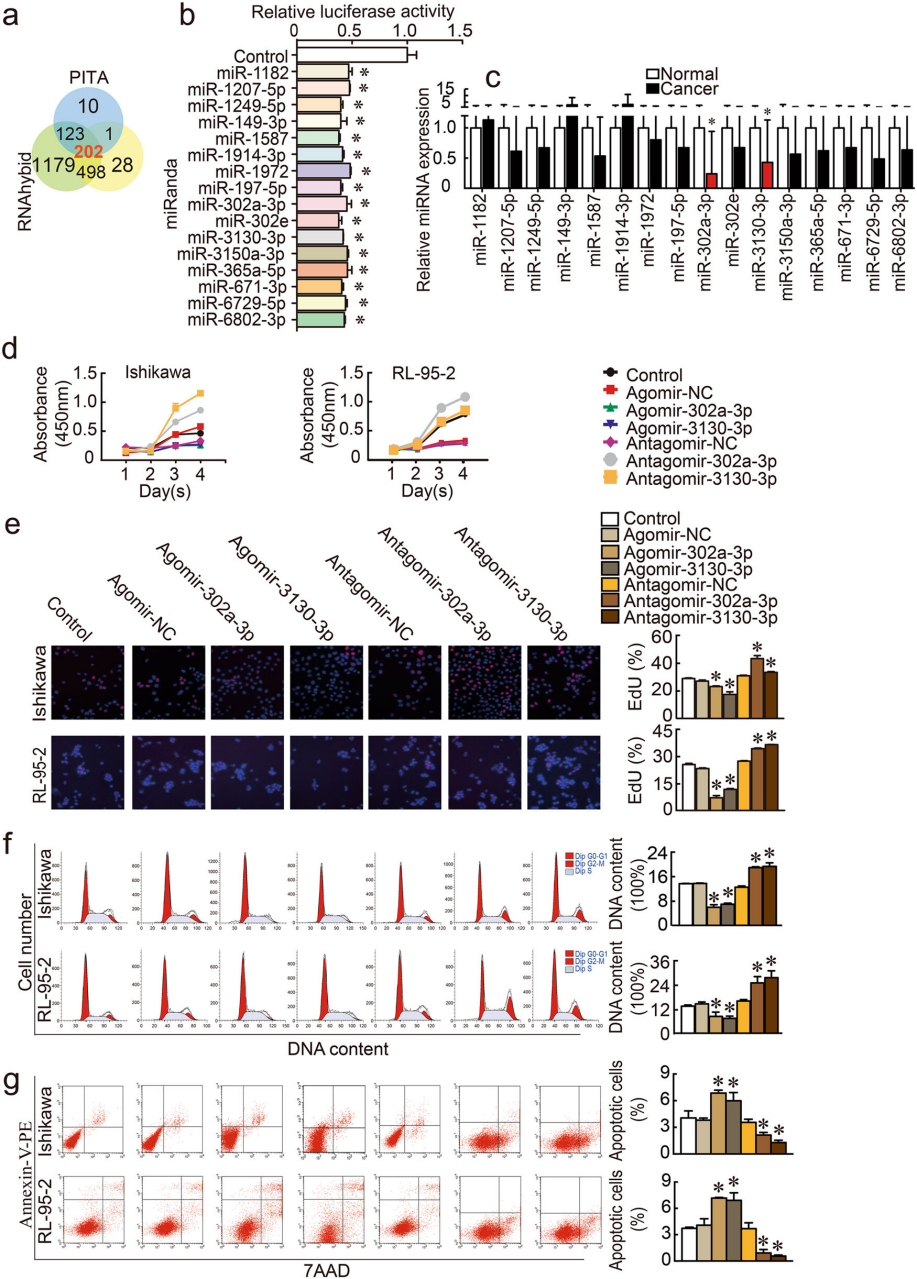

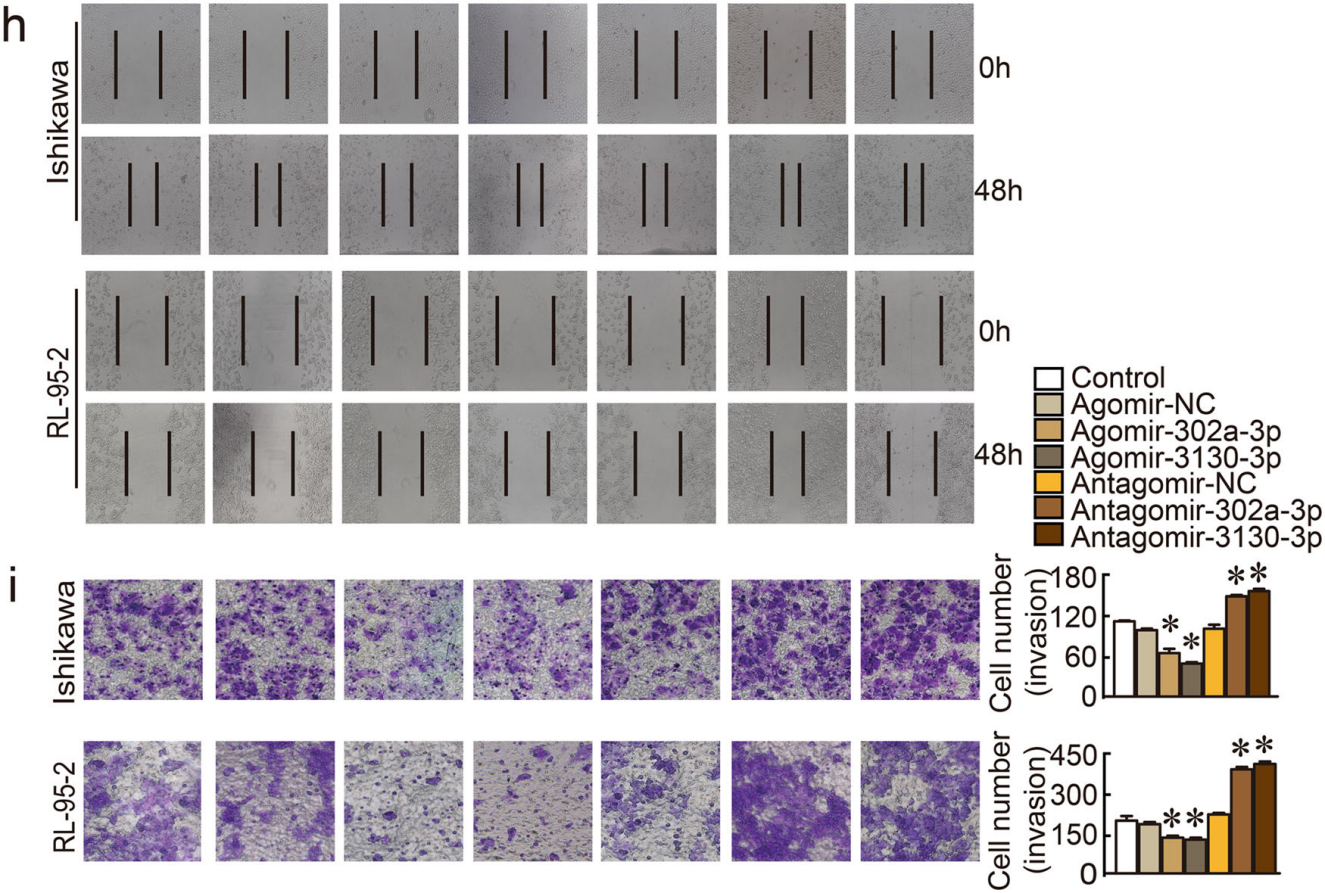
MiR-302a-3p and miR-3130-3p were down-regulated in endometrial cancer tissues and inhibited cell proliferation, migration, and invasion, while promoting apoptosis of human Ishikawa and RL-95-2 cell lines . **a** A total of 202 miRNAs were predicted by bioinformatics programs to putatively interact with LINC01016. **b** Among the 202 miRNAs co-transfected with the reporter vector LINC01016-WT in HEK293T cells, the relative luciferase activities of 16 miRNAs were most inhibited. **c** Expression levels of 16 miRNAs in endometrial cancer tissues and normal tissues (33 cases in cancer group and 20 cases in normal group). **d**, **e** Inhibitory effect of miR-302a-3p/miR-3130-3p on the proliferation of Ishikawa and RL-95-2 cell lines, as shown by CCK-8 and EdU assays. **f** Representative flow cytometry images depicting the cell cycle phases of Ishikawa and RL-95-2 lines with the transfection status of miR-302a-3p/miR-3130-3p indicated. **g** Representative flow cytometry images of apoptotic Ishikawa and RL-95-2 cell lines with the transfection status of miR-302a-3p/miR-3130-3p indicated. **h** Wound healing assays were performed to investigate cell migration capacity. **i** Transwell assays were carried out to investigate the numbers of invading cells in response to altered expression of miR-302a-3p/miR-3130-3p. Representative images and accompanying statistical plots are presented. Data are presented as the means ± SD from three independent experiments. *P < 0.05

The results of the three bioinformatics prediction programs (PITA, miRanda, and RNAhybid) identified 202 miRNAs that potentially interacted with LINC01016 (Fig. 2a). We then co-transfected HEK293T cells with a wild-type (WT) dual-luciferase LINC01016 construct and these 202 miRNAs and performed the dual-luciferase assay. We found that 16 of the candidate miRNAs exhibited strong interaction with LINC01016 (Fig. 2b). The luciferase activities of other 186 miRNAs are shown in Supplementary Figure S3. The expression levels of the 16 miRNAs were detected in endometrial cancer tissues and normal tissues using qRT-PCR. Among the miRNAs, miR-302a-3p and miR-3130-3p were significantly down-regulated in cancer tissues, compared with normal tissues by qRT-PCR (Fig. 2c) and ISH (Supplementary Figure S1b). In addition, we analyzed whether the expression of LINC01016 and miR-302a-3p/miR-3130-3p were correlated (Supplementary Figure S4) and evaluated the relationships of these components with clinical pathological parameters (Supplementary Table S1).

Ectopic expressions of miR-302a-3p/miR-3130-3p were tested by qRT-PCR (Supplementary Figures S2b and c) and the dysregulation of miR-302a-3p/miR-3130-3p had the opposite effect on ectopic LINC01016 expression. CCK-8, EdU, flow cytometry, wound healing assays, and transwell assays were carried out to examine changes in the biological behaviors resulting from altered miR-302a-3p and miR-3130-3p expression. Our findings indicated that increased expression of miR-302a-3p/miR-3130-3p decreased the proliferative ability of cells (Fig. 2d, e). In cells transfected with agomir, the proportion of endometrial cancer cells in G2–M phase decreased. In contrast, antagomir increased the number of cells in G2–M phase (Fig. 2f). The proportion of total apoptotic cells significantly increased in response to overexpression of miR-302a-3p/miR-3130-3p (Fig. 2g). Upregulation of miR-302a-3p/miR-3130-3p interfered with the migration of cells from a monolayer (Fig. 2h) and attenuated cell invasion (Fig. 2i).

### MiR-302a-3p and miR-3130-3p have moderate affinity for LINC01016 and mediate the tumor-promoting effect of LINC01016 on endometrial cancer cells

**Fig. 3:**
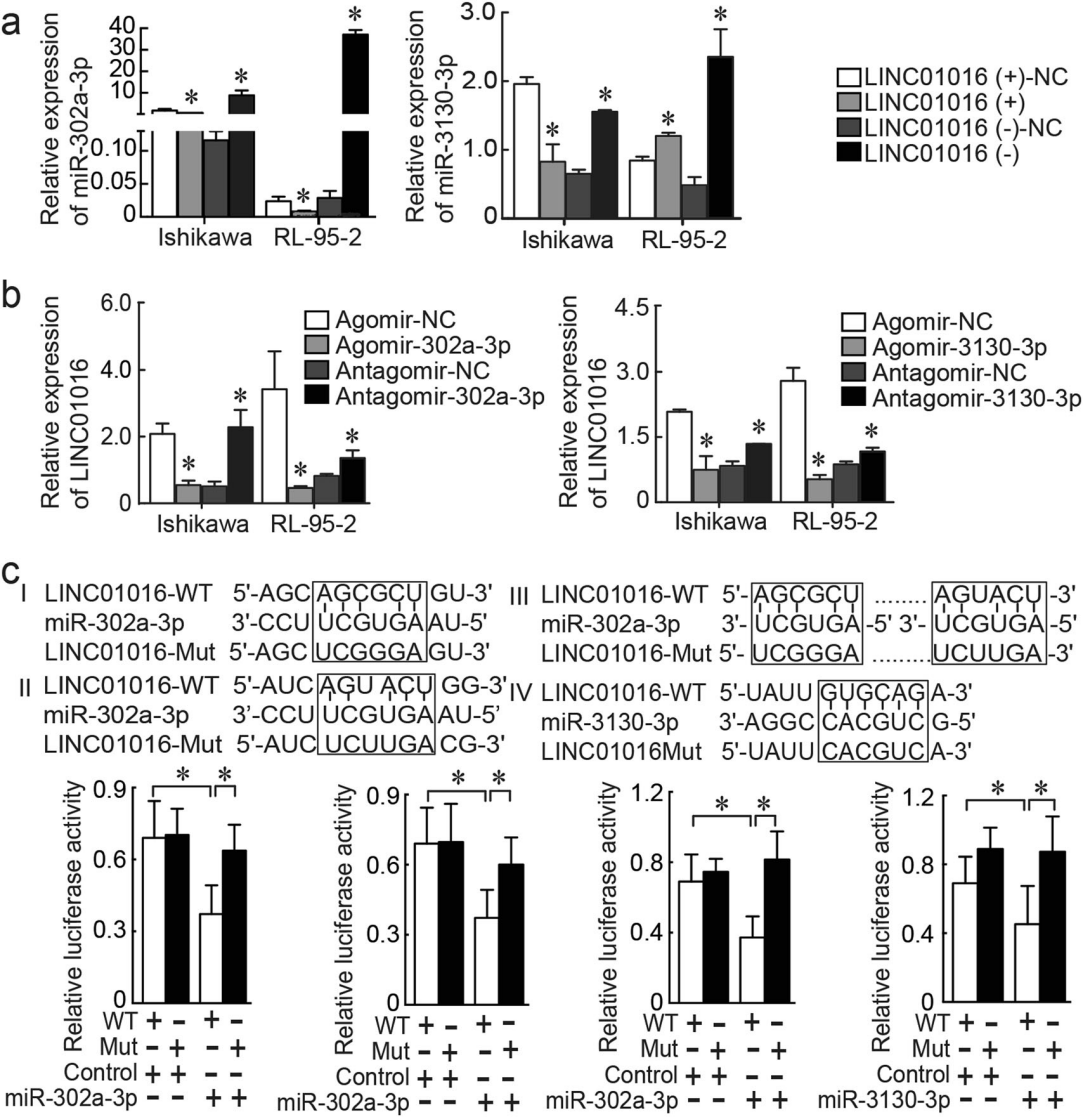
MiR-302a-3p and miR-3130-3p possessed binding sites for LINC01016 in endometrial cancer cells. **a** Relative expression levels of miR-302a-3p/miR-3130-3p were determined with qRT-PCR after transfection with lentiviral constructs harboring LINC01016 (+), LINC01016 (−), and scrambled controls (NC). **b** Relative expression levels of LINC01016 by qRT-PCR after cells were transfected with agomir-302a-3p, antagomir-302a-3p, or NC. **c** Luciferase assays were carried out for miR-302a-3p/miR-3130-3p and LINC01016. Predicted miR-302a-3p/miR-3130-3p binding sites in LINC01016 and the designed mutant sequence are shown. Data are presented as the means ± SD from three independent experiments. *P < 0.05

Considering recent research describing the role of ceRNAs in neoplasia, we hypothesized that LINC01016 might function as a ceRNA in endometrial cancer by competitively binding with miR-302a-3p/miR-3130-3p and promoting the downstream degradation of mRNAs. To evaluate this hypothesis, we performed qRT-PCR in cells post-transfected with LINC01016, by which we determined that miR-302a-3p/miR-3130-3p expression was down-regulated in the LINC01016 (+) group, compared with the LINC01016 (+)-NC group. In contrast, miR-302a-3p/miR-3130-3p expression was upregulated in the LINC01016 (−) group, compared with the LINC01016 (−)-NC group (Fig. 3a). When miR-302a-3p/miR-3130-3p was overexpressed by agomir, LINC01016 expression was down-regulated; when miR-302a-3p/miR-3130-3p was knocked down by antagomir, LINC01016 expression was upregulated (Fig. 3b). The results of bioinformatics predictions indicated two sites on LINC01016 that potentially could interact with miR-302a-3p, and one site that could interact with miR-3130-3p (Fig. 3c). Luciferase assays were conducted to ascertain whether LINC01016 could directly target miR-302a-3p/miR-3130-3p via these putative binding sites. The luciferase activity of the miR-302a-3p/miR-3130-3p and LINC01016 WT co-transfected group was substantially diminished compared with the miR-302a-3p/miR-3130-3p-NC and LINC01016 WT co-transfected group. In contrast, miR-302a-3p/miR-3130-3p did not affect the luciferase activity in the LINC01016 Mut group (Fig. 3c).

In addition, we performed the co-transfection of LINC01016 and miR-302a-3p/miR-3130-3p, which verified that miR-302a-3p/miR-3130-3p functionally reversed the tumor-promoting effect of LINC01016 on cell proliferation, cell cycle regulation, apoptosis, migration, and invasion. As shown in Supplementary Figure S5, LINC01016 (−) cells co-transfected with agomir-302a-3p/miR-3130-3p had the strongest inhibitory effect on malignant biological behavior. Collectively, LINC01016 could function as a ceRNA by sequestering miR-302a-3p/miR-3130-3p, which could restore the translation of target mRNAs. Conversely, miR-302a-3p/miR-3130-3p could regulate the expression of LINC01016, forming two negative feedback loops, and influence the malignant phenotype of endometrial cancer cells.

### NFYA and SATB1 were involved in the LINC01016-miR-302a-3p/miR-3130-3p-induced malignant progression of endometrial cancer cells

**Fig. 4:**
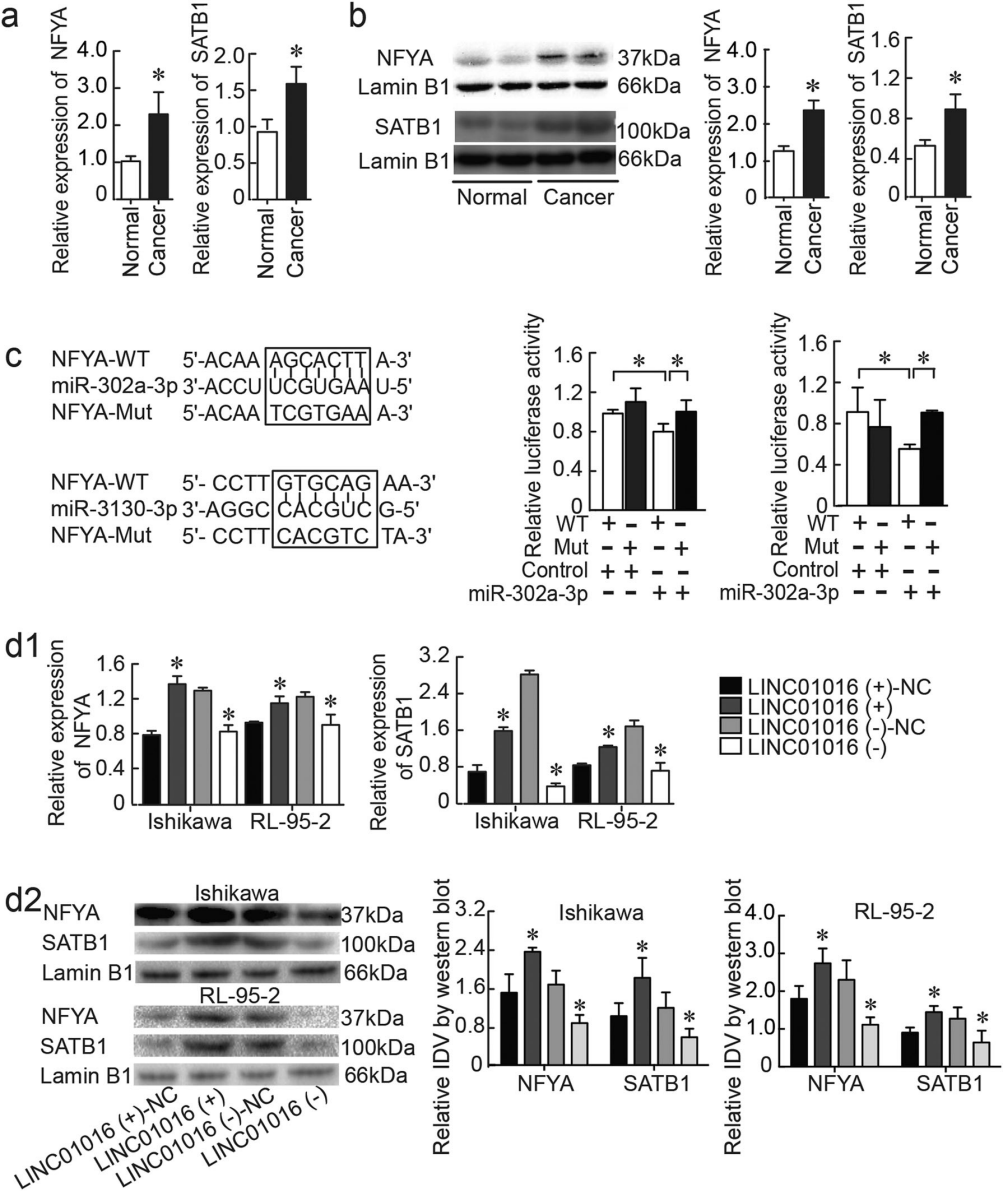

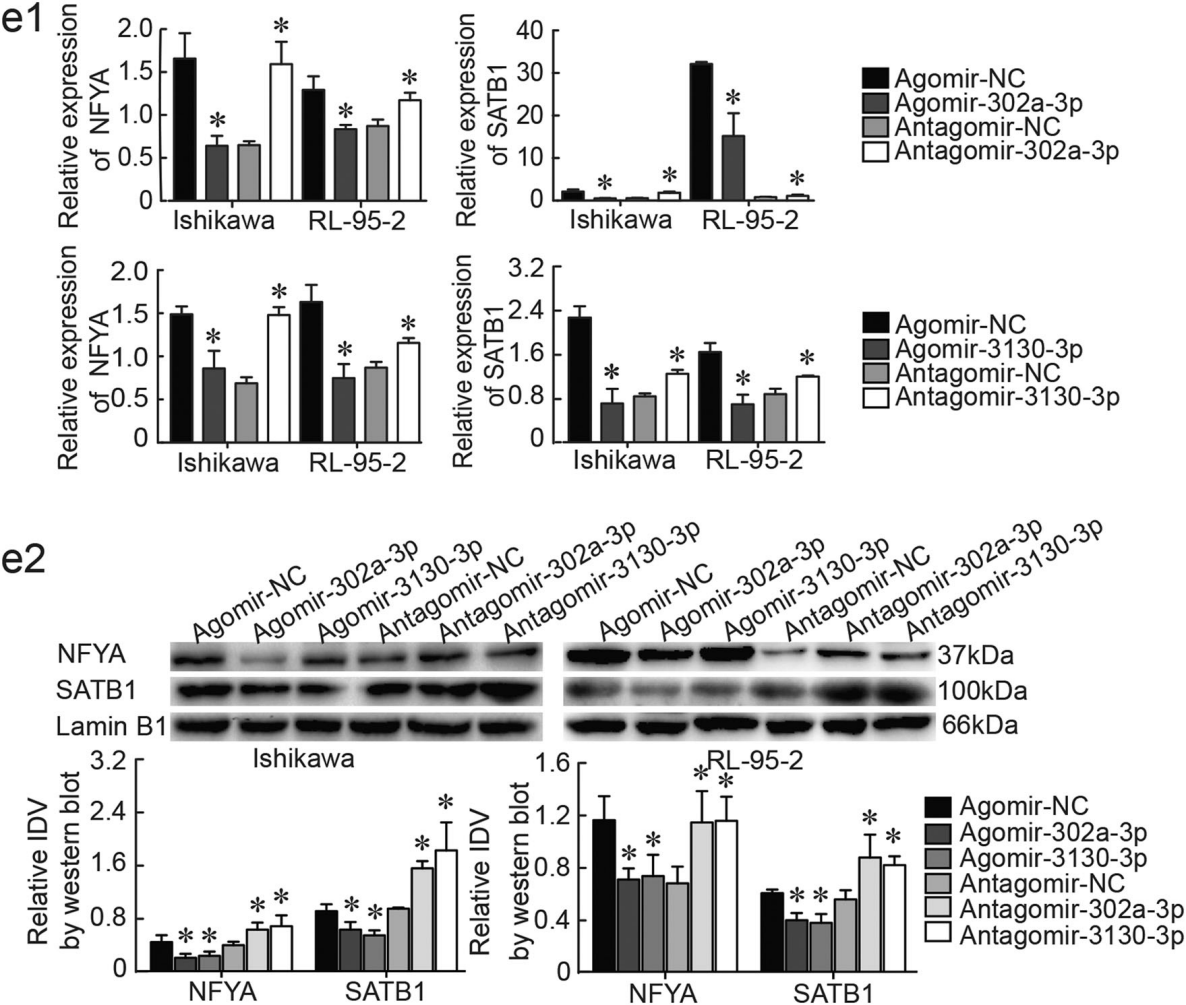
NFYA and SATB1 were involved in the LINC01016-miR-302a-3p/miR-3130-3p regulatory axis **a**, **b** NFYA and SATB1 were overexpressed in endometrial cancer tissues, as shown by qRT-PCR and western blot (33 cases in cancer group and 20 cases in normal group). **c** The predicted miR-302a-3p/miR-3130-3p binding sites in NFYA and the designed mutant sequence are indicated. A luciferase assay of miR-302a-3p/miR-3130-3p and NFYA was conducted. **d1**, **d2** The results of qRT-PCR and western blot demonstrated that LINC01016 regulated NFYA and SATB1 expression in Ishikawa and RL-95-2 cells. **e1**, **e2** Findings of qRT-PCR and western blot showed that miR-302a-3p/miR-3130-3p regulated NFYA and SATB1 expression in Ishikawa and RL-95-2 cells. Data are presented as the means ± SD from three independent experiments. *P < 0.05

We confirmed that NFYA and SATB1 mRNA and protein were overexpressed in endometrial cancer tissues compared to normal tissues by qRT-PCR and western blotting (Fig. 4a) and immunohistochemistry (IHC) (Supplementary Figure S1c and d). Via TargetScan, we identified NFYA as a downstream gene target of miR-302a-3p and miR-3130-3p. To validate a direct interaction between miR-302a-3p/miR-3130-3p and NFYA, we inserted the full-length 3’-UTR of NFYA into a luciferase reporter construct (pmirGLO). Transfection of miR-302a-3p/miR-3130-3p significantly reduced luciferase activity of the reporter carrying the NFYA sequence. This down-regulation could be abolished by transfection of the mutated form of NFYA, indicating that miR-302a-3p and miR-3130-3p bind to the 3’-UTR of NFYA, specifically (Fig. 4c).

To explore whether LINC01016 could function as a ceRNA for NFYA by modulating miR-302a-3p/miR-3130-3p in endometrial cancer, NFYA and SATB1 levels were measured by qRT-PCR and western blot in cells transfected with LINC01016 (+), LINC01016 (−), agomir-302a-3p/miR-3130-3p, or antagomir-302a-3p/miR-3130-3p. Overexpression of LINC01016-induced expression of NFYA and SATB1 mRNA and protein relative to the LINC01016 (+)-NC group; inhibition of LINC01016 expression had the opposite effects (Fig. 4d1, d2). Overexpression of miR-302a-3p/miR-3130-3p significantly reduced both the mRNA and protein expression levels of NFYA and SATB1, compared to the agomir-NC group. Inhibition of miR-302a-3p/miR-3130-3p expression had the opposite effects (Fig. 4e1, e2). Moreover, the expression levels of NFYA and SATB1 in each co-transfection group are depicted in Supplementary Figure S6. Cells co-transfected with LINC01016 (+) and antagomir-302a-3p/miR-3130-3p exerted the highest NFYA level while cells co-transfected with LINC01016 (−) and agomir-302a-3p/miR-3130-3p showed the lowest. Collectively, our results illustrated that LINC01016-miR-302a-3p/miR-3130-3p-dependent regulation modulated the expression of NFYA and SATB1 in endometrial cancer cells. Taken together, our findings suggested that LINC01016 and miR-302a-3p/miR-3130-3p regulated the expression of downstream NFYA and SATB1 by means of reciprocal interaction.

### NFYA promotes tumorigenesis in endometrial cancer cells

**Fig. 5:**
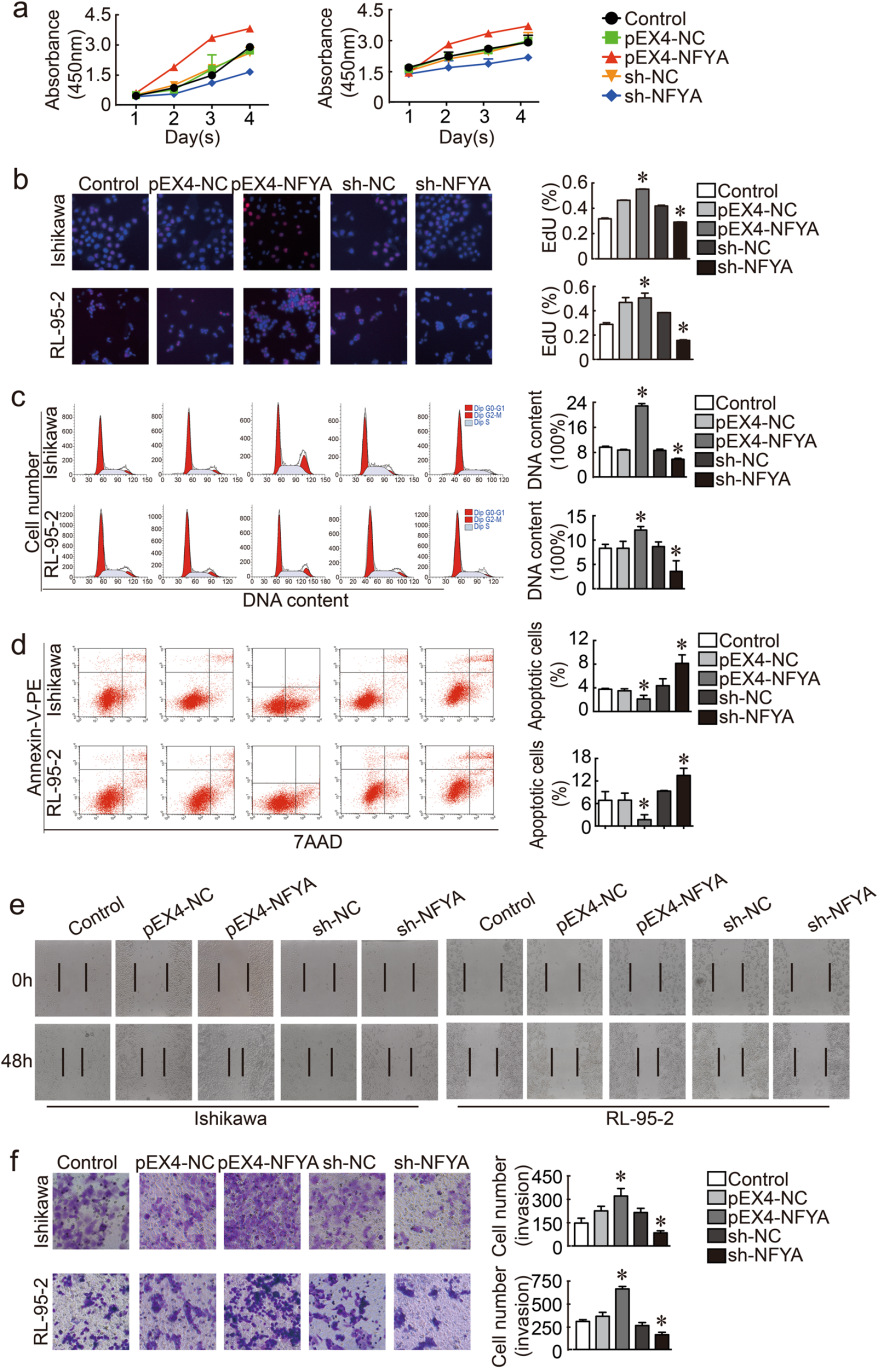
NFYA was a pro-oncogene in human Ishikawa and RL-95-2 cell lines. **a**, **b** Auxo-effect of NFYA on the proliferation of Ishikawa and RL-95-2 cell lines, as depicted by CCK-8 and EdU assay results. **c** Representative flow cytometry results of the cell cycle phases of Ishikawa and RL-95-2 cell lines with transfection of NFYA are shown. **d** Representative flow cytometry findings of apoptotic Ishikawa and RL-95-2 cell lines are shown with transfection status of NFYA indicated. **e** Wound healing assays were carried out to investigate the cell migration capacity. **f** Transwell assays were performed to investigate the quantities of invading cells when NFYA expression was altered. Representative images and accompanying statistical plots are presented. Data are presented as the means ± SD from three independent experiments. *P < 0.05

Given that NFYA and SATB1 were found to be overexpressed in endometrial cancer tissues, we explored the role of NFYA in malignant progression in endometrial carcinogenesis. The results of CCK-8 and EdU assays indicated that cell proliferation was increased marginally by overexpression of NFYA (Fig. 5a, b). Overexpression of NFYA increased the proportion of endometrial cancer cells in G2–M phase. However, knockdown of NFYA decreased the quantity of cells in G2–M phase (Fig. 5c). The proportion of total apoptotic cells was significantly decreased in response to increased expression of NFYA (Fig. 5d). Increased expression of NFYA also facilitated cell invasion (Fig. 5e) and migration (Fig. 5f). We concluded that NFYA played a positive role in regulation of the malignant phenotype of endometrial cancer cells via the LINC01016-miR-302a-3p/miR-3130-3p axis.

### NFYA exerts tumor-promoting effects on endometrial cancer cells by directly targeting SATB1

**Fig. 6:**
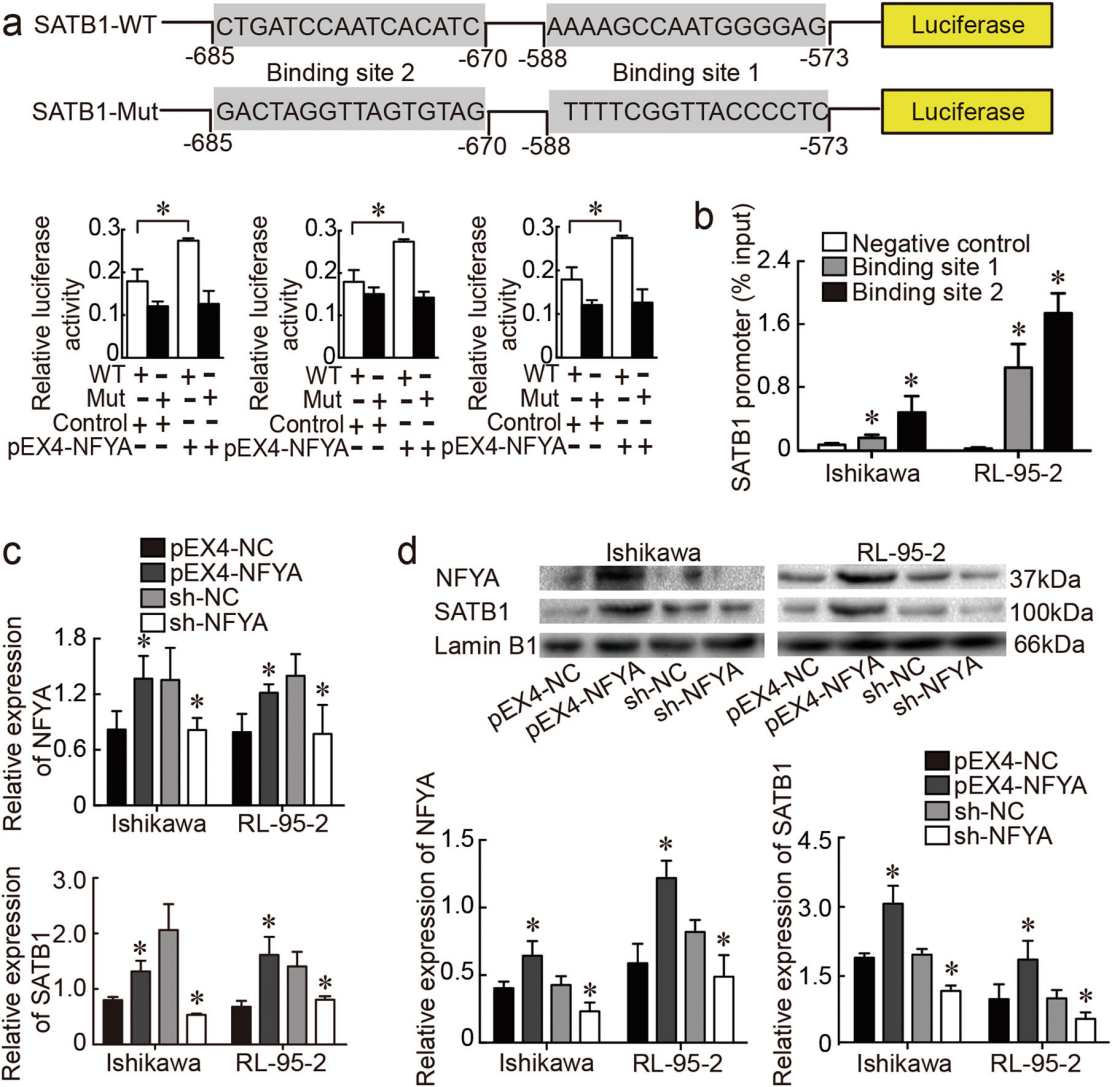
SATB1 was positively regulated by NFYA. **a** The predicted NFYA binding sites in the promotor of SATB1 and the designed mutant sequence are indicated. A luciferase assay of NFYA and SATB1 was carried out. **b** ChIP assays were performed in Ishikawa and RL-95-2 cells followed by qRT-PCR. **c** Results of qRT-PCR confirmed that NFYA regulated SATB1 expression in Ishikawa and RL-95-2 cells. **d** The results of western blots showed that NFYA regulated SATB1 expression in Ishikawa and RL-95-2 cells. Data are presented as the means ± SD from three independent experiments. *P < 0.05

We next investigated the relationship between NFYA and SATB1. Specifically, potential binding sites for NFYA on the SATB1 promoter were predicted from the JASPAR database (http://jaspar.genereg.net/cgi-bin/jaspar_db.pl) (Fig. 6a). The common promotor region of SATB1 was subcloned into a luciferase reporter construct. Assay results indicated that luciferase activity in the SATB1-WT group was significantly increased after transfection with the pEX4-NFYA plasmid, compared to pEX4-NC. Luciferase activity in the SATB1-Mut group was decreased in cells transfected with pEX4-NFYA and its corresponding negative control construct (Fig. 6a). We next performed chromatin immunoprecipitation (ChIP) assays to assess the transcriptional regulation of NFYA on the two potential binding sites of the SATB1 promotor region. Ultrasound lysis of the cell yielded evenly distributed DNA fragments ranging from 200 to 1000 base pairs (bp) (Supplementary Figure S7). The results of qRT-PCR indicated that two sites acted as targets of NFYA (Fig. 6b). Expression levels of SATB1 subsequently were evaluated by qRT-PCR and western blot in cells transfected with pEX4-NFYA, sh-NFYA, and the respective scrambled negative controls. SATB1 mRNA was increased in the pEX4-NFYA group, as was SATB1 protein expression (Fig. 6c, d). Collectively, these results suggested that NFYA contributed to the malignant behavior of endometrial cancer cells by directly binding to the promotor region of SATB1.

### LINC01016 down-regulation combined with miR-302a-3p/miR-3130-3p overexpression significantly inhibits the malignant tumor phenotype of endometrial cancer cells in vivo

**Fig. 7:**
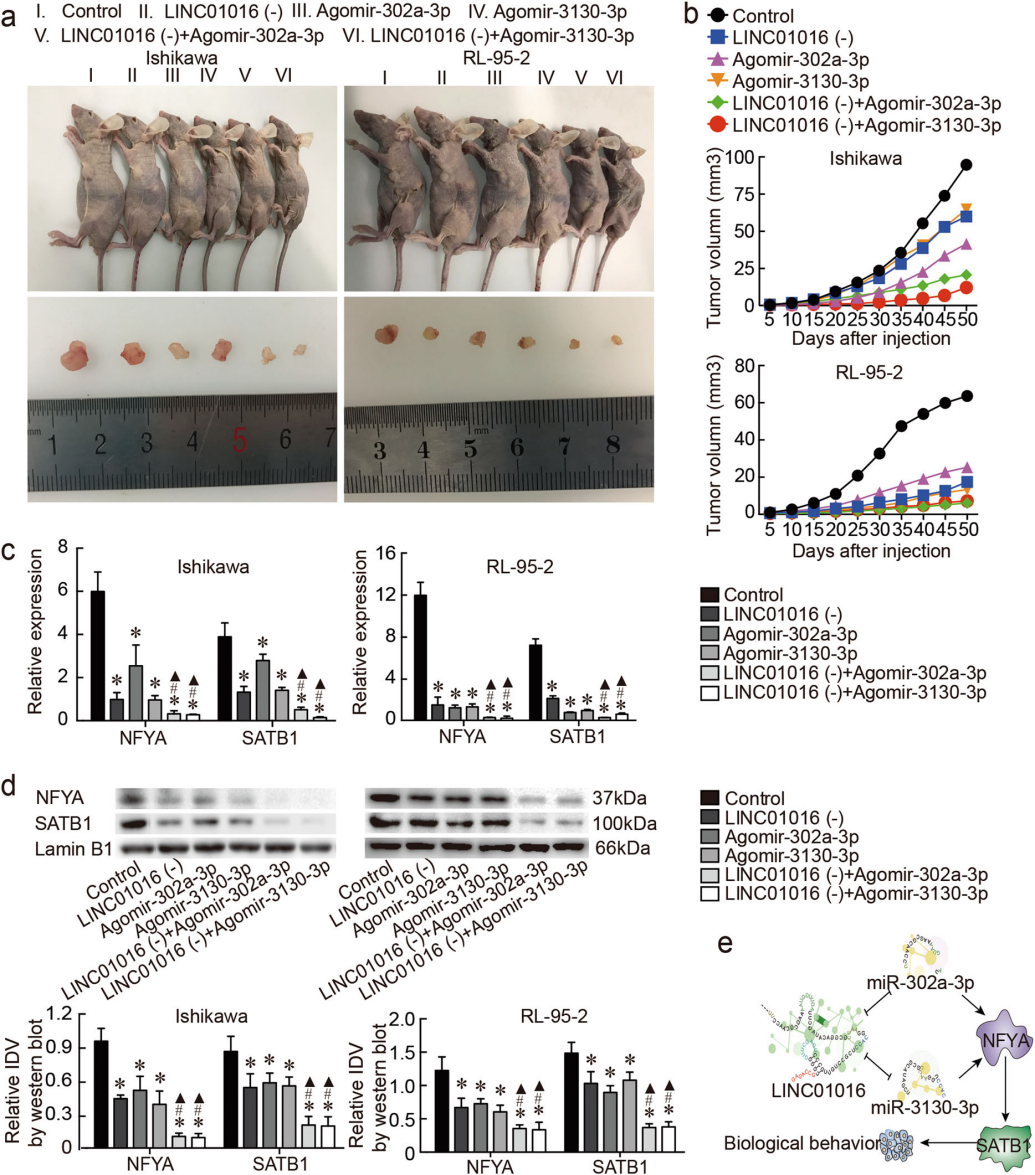
In vivo study of tumor xenografts. **a** Nude mice harboring tumors, and a specimen from each respective group, are shown. **b** Tumor widths and lengths were determined and tumor volume was calculated for each animal at 5-day intervals after injection, in total 10 times. **c**, **d** NFYA and SATB1 expression levels as determined by qRT-PCR and western blot in tumor tissues obtained from each group. **e** The proposed negative feedback loop mechanism underlying the LINC01016-miR-302a-3p/miR-3130-3p/NFYA/SATB1 axis in Ishikawa and RL-95-2 cells. Data are presented as the means ± SD from three independent experiments. *P < 0.05

We ascertained the influence of LINC01016 knockdown, miR-302a-3p/miR-3130-3p overexpression, and their combined effects on endometrial tumors in nude mice. Tumor volume in the co-transfected group was smaller than in the LINC01016 (−) group or the antagomir-302a-3p/miR-3130-3p group for both Ishikawa and RL-95-2 cells (Fig. 7a, b). Similarly, the expression levels of NFYA and SATB1—detected by qRT-PCR and western blot—were lowest in the co-transfected group, compared with the LINC01016 (−) group and the agomir-302a-3p/miR-3130-3p group (Fig. 7c, d). Moreover, ISH and IHC were performed to detect the expression of LINC01016, miR-302a-3p/miR-3130-3p, NFYA, and SATB1 in xenograft tumor tissues formed by Ishikawa cells (Supplementary Figure S1 e-i).

## Discussion

Our study verified for the first time that the expression of the lncRNA LINC01016 was significantly elevated in endometrial cancer tissues and could promote the malignant biological behaviors of endometrial cancer cells. In contrast, miR-302a-3p and miR-3130-3p were down-regulated in endometrial cancer tissues and played a tumor-suppressive role. Interestingly, miR-302a-3p and miR-3130-3p possessed binding sites for LINC01016 and formed two reciprocal negative feedback loops. Overexpression of LINC01016 together with miR-302a-3p/miR-3130-3p knockdown exhibited the most evident tumorigenic effect. Furthermore, NFYA was elevated by LINC01016 via the impairment of miR-302a-3p/miR-3130-3p expression, both of which directly target the 3′-UTR of NFYA. With a high expression level in endometrial cancer tissues, NFYA directly targeted the promotor region of SATB1 and contributed to the transcription of SATB1. Remarkably, our in vivo study robustly demonstrated the inhibition of LINC01016 with the restoration of miR-302a-3p/miR-3130-3p exerted the lowest tumorigenic ability in nude mice.

Mounting evidence has indicated that lncRNAs play a crucial part in numerous biological events^28^. For example, significantly reduced expression of the lncRNA-LOWEG was found in gastric cancer tissues and cell lines compared to that in adjacent tissues. LOWEG inhibited cell invasion by upregulating the LIFR gene^29^. GPC3-AS1 promoted hepatocellular carcinoma progression via epigenetically activating GPC3, and was correlated with α-fetoprotein level, tumor size, microvascular invasion, tumor encapsulation, cancer stage, and worse prognosis. Therefore, GPC3-AS1 was identified as a potential therapeutic target for hepatocellular carcinoma^30^. Herein, we found that LINC01016 was overexpressed in endometrial cancer tissues, consistent with LINC01016 expression in breast cancer. Upregulation of LINC01016 promoted endometrial cell proliferation, migration, and invasion and inhibited apoptosis. Therefore, we postulated that LINC01016 was a putative oncogene in endometrial cancer.

Emerging evidence has confirmed the abundant functional patterns of miRNAs^31^. MiR-326 is regulated by the phosphatidylinositide 3-kinase pathway. Overexpression of miR-326 resulted in significantly reduced proliferation, colony formation suppression, and hindered the migration capacity of glioma cells^32^. In contrast, miR-221 acted as an oncogene in non-small-cell lung carcinoma, and upregulation of miR-221 promoted the malignant biological behavior of SPCA1 and H1299 cell lines by directly targeting TIMP2 at both the mRNA and protein levels^33^. Cyclin D1 was activated during embryonic stem cell (ESC) differentiation in a germ cell nuclear factor (GCNF)-dependent way by inhibiting the expression of miR-302a^34^. In addition, miR-302a may be a potential biomarker for osteosarcoma since it can inhibit osteosarcoma cell growth and metastasis by targeting ADAM9^35^. 5‑fluorouracil (5-FU)-induced cell death and viability was inhibited by insulin‑like growth factor-1 receptor (IGF-1R), which possessed a binding site for miR-302a^36^. However, there is still no research regarding miR-3130-3p. We examined that miR-302a-3p and miR-3130-3p were down-regulated in endometrial cancer tissues and inhibited the malignant behavior of cancer cells experimentally. By restraining the proliferation, migration, and invasion of cancer cells and by promoting their apoptosis, miR-302a-3p and miR-3130-3p may represent two potential therapeutic targets in endometrial cancer treatment. However, the underlying mechanisms still need to be explored.

The model of lncRNA functioning as ceRNAs to shield critical target genes from miRNA-mediated repression is well established^37–40^. Since a sequestration-feedback regulatory model between miRNAs and mRNAs has been widely accepted^41^, this anti-correlated dynamic model has been described for lncRNAs and miRNAs. Bartel and colleagues^42,43^ were the first to postulate that lncRNAs competitively bind to miRNAs through miRNA response elements and attenuate the binding of miRNAs to the downstream 3′-UTR, 5′-UTR, or coding regions of mRNAs. Together with protein-coding genes and pseudogenes, lncRNAs are considered ceRNAs^44^. Ectopic expression of certain miRNAs can alter their targeting of lncRNAs, thereby forming an interoperable cycle^45^. Jiemei et al. found that overexpression of miR-145 suppressed expression of the lncRNA TUG1, whereas knockdown of TUG1 elevated miR-145 expression; this reciprocal repression was illustrated by physical interaction studies in bladder cancer cells. In addition, the decreased invasiveness of bladder cancer cells induced by knocking down TUG1 could be rescued by inhibiting miR-145^46^. KCNQ1OT1 promoted glioma cell progression via decreasing miR-370 expression and miR-370 inversely modulated KCNQ1OT1 expression. Luciferase reporter assays confirmed the miR-370 binding site in KCNQ1OT1 and RNA immunoprecipitation assays supported the hypothesis of KCNQ1OT1 and miR-370 functioning together in a RNA-induced silencing complex (RISC) complex^47^. LncRNA RSU1P2 promoted the malignant phenotype of cervical carcinoma cells and regulated the expression of N-myc by acting as a ceRNA via competition for the shared miRNA let-7a. The transcriptional factor N-myc in turn activated RSU1P2 expression and thus formed a positive feedback loop with RSU1P2, which could have strong benefits^48^. In recent years, a miRNA-mediated double-negative feedback loop was proposed to explain how miRNAs could function as a bistable switch without cooperatively binding with transcriptional factors^49^. To evaluate these features in LINC01016, we first predicted binding sites for miR-302a-3p and miR-3130-3p using three bioinformatics software programs (PITA, miRanda and RNAhybid) and verified these results by luciferase assays. Further, LINC01016 negatively regulated miR-302a-3p/miR-3130-3p and miR-302a-3p/miR-3130-3p in turn inhibited LINC01016. Knockdown of LINC01016 in company with miR-302a-3p/miR-3130-3p overexpression significantly restrained the malignant phenotype of cancer cells in vitro and ablated tumor growth in vivo. Taken together, these results led to the first suggestion that two negative feedback loops function in the biological behavior of endometrial cancer cells. In this context, once the dynamic equilibrium between LINC01016 and miR-302a-3p/miR-3130-3p is disrupted, the negative feedback process will amplify the associated gene or biological phenomenon and trigger a perpetual cycle of auto-enhancement. This may explain, at least in part, why cancer exhibits inexhaustible survivability and may lend insights into cancer diagnostics and therapeutics^50,51^. To our knowledge, this study is the first to find such a collaboration and elaborate the underlying mechanism between lncRNA and miRNAs in endometrial cancer.

Numerous cell events are mediated by the binding of miRNAs to the 3′-UTR of target genes. For example, miR-4530 could directly target the 3′-UTR of VASH1 and promote angiogenesis, inhibit proliferation, and induce apoptosis by suppressing the expression of VASH1 in breast carcinoma cells^52^. MiR-138 inhibited osteosarcoma cell proliferation and invasion via direct targeting of SIRT1, which suggested that the miR-138/SIRT1 axis could represent a promising therapeutic target for osteosarcoma^53^. NFYA is a transcriptional factor that recognizes the CCAAT motif. Together with its other two subunits, NFYB and NFYC, NFYA is involved in multiple aspects of cell proliferation and differentiation^23,54^. There are two isoforms (long isoform and short isoform) of NFYA, and their positive promotor-binding activities might be different in various cell types^55^. Although other investigators determined that NFY regulated ALDH1A1 in cooperation with Oct-1 in endometrial adenocarcinoma^24^, the function of this molecule as a transcriptional factor in endometrial cancer has not been explored previously. In our study, NFYA expression was elevated in endometrial cancer tissues and inhibition of NFYA hindered cancer cell progression. NFYA was confirmed to be a target of miR-302a-3p and miR-3130-3p and introduction of miR-302a-3p/miR-3130-3p decreased the LINC01016-induced overexpression of NFYA, which proved that NFYA was involved in the LINC01016- miR-302a-3p/miR-3130-3p regulation axis.

SATB1 can facilitate tumorigenesis and progression of cancer^56,57^, and was recognized as a biomarker with high prognostic significance in several cancers^58,59^ including endometrial cancer^60^. However, the specific mechanical role of SATB1 in endometrial cancer remains ambiguous. Our group previously hypothesized that the promotor region of SATB1 could bind with NFYA and the interaction of NFYA and SATB1 was confirmed by luciferase and ChIP assays. In the current study, overexpression of SATB1 was analogous to NFYA upregulation. We determined that LINC01016 favored NFYA, and thus SATB1 accumulation, by repressing miR-302a-3p and miR-3130-3p expression. Hence, SATB1 was identified as a downstream gene target in LINC01016-miR-302a-3p/miR-3130-3p-NFYA regulatory axis in endometrial cancer.

Our study first revealed that LINC01016 promoted the malignant biological behavior of endometrial cancer cells by directly inhibiting miR-302a-3p and miR-3130-3p. In addition, miR-302a-3p and miR-3130-3p could inversely decrease LINC01016 expression. MiR-302a-3p and miR-3130-3p restrained tumor progression by decreasing expression of downstream gene NFYA, which could have acted as a transcriptional factor for SATB1. LINC01016 and miR-302a-3p/miR-3130-3p made up two bidirectional regulatory feedback loops that mediated the malignant phenotype of endometrial cancer cells via NFYA and its target SATB1 (Fig. 7e). The LINC01016/miR-302a-3p/miR-3130-3p/NFYA/SATB1 axis highlighted a novel class of lncRNA–miRNA interactions, which may guide future treatments in endometrial cancer.

## Materials and methods

### Bioinformatics prediction

We applied three bioinformatics software programs to predict miRNAs with potential binding sites on LINC01016: miRanda, PITA, and RNAhybrid (Ribobio Co., Ltd, Guangzhou, China). The results of each program were screened, and common miRNAs were selected as candidate LINC01016-interacting miRNAs.

### Patients and specimens

A total of 33 endometrial cancer tissues and 20 normal endometrial tissues were obtained from patients at the Department of Obstetrics and Gynaecology, Shengjing Hospital of China Medical University, China, from 2011 to 2015. Normal tissues were collected from women who underwent a hysterectomy or endometrial curettage for endometrial-irrelevant diseases. All patients provided informed consent, and this study was approved by the Ethics Committee of Shengjing Hospital of China Medical University. Histological diagnosis and tumor grade were assessed by three experienced pathologists in accordance with the International Federation of Gynaecology and Obstetrics (FIGO 2009). No patient received local or systemic treatment preoperatively.

### Cell culture

Ishikawa and RL-95-2 endometrial cell lines were maintained in RPMI medium 1640 (Bioind, Kibbutz Beit Haemek, Israel) and Dulbecco's modified Eagle's medium (DMEM)/F12 medium (Bioind) (with 1% insulin added), respectively. Cultures were supplemented with 10% fetal bovine serum (FBS), 50 IU/mL penicillin, and 50 mg/mL streptomycin (Invitrogen, Carlsbad, CA). The human embryonic kidney cell line HEK293T was maintained in DMEM/high-glucose medium (HyClone) supplemented with 10% FBS. Cells were maintained at 37 °C in a humidified incubator in the presence of 5% CO_2_.

### Transfection of cells

Lentivirus overexpression plasmids harboring LINC01016 (LV5-LINC01016) were purchased from GeneChem (Shanghai, China) and were transfected at a multiplicity of infection (MOI) of 10. Lentivirus low-expression plasmids harboring shLINC01016 (GV248-LINC01016) were purchased from GenePharma (Shanghai, China) and were transfected at an MOI of 50. Transfection of miRNAs against NFYA was achieved with a Lipofectamine 3000 kit (Invitrogen) according to the manufacturer’s instructions. The agomir, antagomir, and their respective scrambled negative control RNAs were purchased from GenePharma. The NFYA overexpression plasmid (pEX4-NFYA), NFYA low-expression plasmid (sh-NFYA), and their respective negative control DNAs were purchased from GenePharma. The sequences of the plasmids and RNA oligo/inhibitor are listed in Supplementary Table S2.

### RNA extraction and qRT-PCR

Total RNA was extracted from tissues and cells using TRIzol reagent (Takara, Dalian, China) based on the manufacturer’s instructions. The complementary DNAs (cDNAs) corresponding to the lncRNAs and mRNAs of interest were reverse-transcribed from 1 μg of total RNA using PrimeScript RT-polymerase (Takara). The cDNAs for the miRNAs of interest were synthesized using Mir-X^Tm^ miRNA First-Strand Synthesis (Clontech, Dalian, China). The qRT-PCR was performed using SYBR-Green Premix (Takara) with specific PCR primers (Sangon Biotech Co., Ltd, Shanghai, China). Glyceraldehyde-3-phosphate dehydrogenase (GAPDH) and RNU6 (U6) were used as internal controls due to their stability across all groups studied. Fold changes were calculated by means of the 2^−ΔΔCt^ method. Primer sequences are listed in Supplementary Table S3.

### Protein extraction and western blotting

Nuclear protein was extracted with a nuclear and cytoplasmic protein extraction kit (Beyotime Biotechnology, Shanghai, China) following cell harvest. Samples were separated by 8% sodium dodecyl sulfate–polyacrylamide gel electrophoresis and then were transferred to polyvinylidene difluoride membranes (Millipore, USA). The membranes were incubated in diluted primary antibodies against NFYA (1:500, Abcam, Cambridge, UK) and SATB1 (1:1000, BD Biosciences) overnight at 4 °C. The membranes were then visualized using Quantity One imaging software (Bio-Rad, California, USA). Lamin B1 was used as a nuclear internal control (1:2000; Proteintech, Hangzhou, China).

### Immunohistochemical analysis

IHC for NFYA and SATB1 were performed on paraffin sections using the primary antibody against NFYA and SATB1 (1:200, Santa Cruz Biotechnology, Santa Cruz, CA) and a horseradish peroxidase-conjugated goat anti-rabbit antibody (Maixin, Fuzhou, China). 3-amino-9-ethylcarbazole (AEC) or Nitro blue tetrazolium chloride/5-Bromo-4-chloro-3-indolyl phosphate (NBT/BCIP) was used to visualize positive reactions. Area quantification was performed by light microscopy.

5′-TCACCAAAACATGGAAGCACTTA-3′

miR-3130-3p: 5′-TTACCCAGTCTCCGGTGCAGC-3′.

### In situ hybridization

The ISH probe used for detecting LINC01016, miR-302a-3p, and miR-3130-3p labeled digoxin was designed and synthesized by Boster Bio-Engineering Company (Wuhan, China). The probe sequences were designed as LINC01016: 5′-TGTCACAGGCCAAGGGGATAGTTCACCACCTTGTTTTCTC-3′; miR-302a-3p: 5’-TCACCAAAACATGGAAGCACTTA-3’; miR-3130-3p: 5′-TTACCCAGTCTCCGGTGCAGC-3′. ISH was performed using the ISH Kit (Boster) according to the manufacturer’s instructions. Briefly, probes were diluted in hybridization buffer, denatured, and then hybridized overnight at 60 °C. The slides were blocked at 37 °C for 30 min and were visualized with 3,3'-diaminobenzidine reaction. Images were digitally acquired on a microscope.

### Luciferase assay

To screen for a set of miRNAs that interacted strongly with LINC01016, HEK293T cells were co-transfected with 5 nmol of either the 202 identified miRNAs or the scrambled negative controls (Ribobio) along with 100 ng of a dual-luciferase reporter vector carrying the wild-type LINC01016 fragment (pmiR-RB-Report™-LINC01016) (Ribobio) using a Lipofectamine 3000 kit (Invitrogen) according to the manufacturer’s instructions. To assess the presence of an interaction between miR-302a-3p/miR-3130-3p with LINC01016, the mutant version of LINC01016 (Ribobio) was added into the co-transfection system.

The 3′-UTR of NFYA, which contains putative miR-302a-3p/miR-3130-3p binding sequences, was cloned downstream of the luciferase gene in the pmirGLO luciferase vector (Promega, Madison, WI). HEK293T cells were then transfected with this construct along with mimics of miR-302a-3p/miR-3130-3p. The promotor region (2000 bp upstream of ATG) of SATB1 was cloned downstream of luciferase vector PGL4.18 (Promega) and was transfected into HEK293T cells together with pEX4-NFYA. Plasmid pRL-TK (Promega) was included as the internal negative control. At 48 h post transfection, the luciferase assay was conducted using a dual-luciferase reporter assay system (Promega) according to the manufacturer’s protocol.

### ChIP assay

ChIP assays were performed according to the manufacturer’s instructions (Merck Millipore, Danvers, MA). Briefly, Ishikawa and RL-95-2 cells were cultured at 37 °C and 5% CO_2_ and were crosslinked with 37% formaldehyde. For immunoprecipitation, sonicated chromatin solutions were incubated at 4 °C overnight with anti-NFYA antibody (Santa Cruz Biotechnology), anti-RNA polymerase II (positive control), and normal mouse IgG (negative control). Crosslinking was reversed, purified DNA was dissolved in 50 µL elution buffer using primers against relevant promoters, and qRT-PCR was carried out. The results are represented as percentages of the input chromatin. Experiments were performed at least in triplicate.

### Cell proliferation assay

Cells were grown in 96-well plates and aliquots of 10 μL of CCK-8 reagent (Dojindo, Japan) per well were added, and cells were then incubated at 37 °C and 5% CO_2_ for 4 h. The OD450 value for each well was detected with a microplate reader, and each group was assayed in triplicate at daily intervals after consecutive seeding for up to 4 days. We also used an EdU cell proliferation assay kit (Ribobio) to measure cell proliferation. Specifically, 100 μL of EdU at a concentration of 50 μM was added per well, and cells were incubated for 2 h. Cell nuclei were stained with Hoechst at a concentration of 1 µg/mL for 30 min. The proportion of cells that incorporated EdU was determined by fluorescence microscopy.

### Cell invasion assays

Transwell filter inserts (8 μm pore size; Corning, NY) were precoated with Matrigel for 30 min at 37 °C. After incubation for 22 h, cells were fixed with 4% polyoxymethylene for 50 min and were stained with 0.1% crystal violet for 50 min. Cells were then imaged under an inverted fluorescence microscope and an image acquisition system (Nikon, Japan). Each group was analyzed in triplicate.

### Wound healing assay

A 10 µL pipette tip was used to create a wound across the cell monolayer, and cells were grown to approximately 90% confluence. Horizontal linear markings were made to clearly distinguish different areas. Cells protruding from the edge of wound were presumed to possess migration ability and were visualized and photographed under a microscope at daily intervals. Experiments were carried out at least in triplicate.

### Cell cycle and apoptosis analysis

After transfection, cells were harvested at 10^6^ cells per group. Cells were washed in phosphate-buffered saline (PBS) and were fixed in 70% ethanol at 4 °C overnight. Cells were then incubated in PBS containing RNase A (at 1:50 of the system), and DNA was stained with propidium iodide (at 1:100 of the system). The proportions of cells in different phases of the cell cycle were assessed by flow cytometry (BD FACSCalibur, New Jersey, USA).

Cells after transfection were also harvested and double-stained with annexin V–PE/7-AAD (Biolegend, San Diego, CA) away from light for 10 min at room temperature. Subsequently, the proportion of apoptotic cells was assessed by flow cytometry (BD FACSCalibur). Each group was analyzed in triplicate.

### Tumor xenograft implantation in nude mice

Post-transfection cells were also evaluated in vivo. Animal experiments were conducted in strict accordance with a protocol approved by the Administrative Panel on Laboratory Animal Care of the Shengjing Hospital. Twelve 4-week-old BALB/C athymic nude mice were purchased from HFK Bioscience Co., Ltd (Beijing, China). Each batch of cells was divided into 6 groups, and 1 × 10^6^ cells were injected subcutaneously in the axillae of the mice. Tumor volume was calculated according to the following formula: tumor volumes (mm^3^) = length × width^2^/2. Consistent with the Institutional Animal Care and Use Committee standards, mice were killed by cervical dislocation if they showed tumor metastasis, lethargy, loss of ≥20% body weight, or other signs of distress.

### Statistical analysis

Data are expressed as means ± SD of three independent experiments. Statistical analyses were performed with GraphPad Prism 6.0 software (La Jolla, CA) and SPSS version 17.0 software (Abbott Laboratories, Chicago, IL). A two-sided Student’s *t*-test or one-way analysis of variance was used to ascertain differences between the two groups. A chi-square test was used to assess the relationship between expression level and clinical pathological parameters. Spearman’s rank correlation method was used for correlation analyses. A *P* < 0.05 was considered statistically significant.

## Electronic supplementary material

Supplementary Figure S1

Supplementary Figure S2

Supplementary Figure S3

Supplementary Figure S4

Supplementary Figure S5

Supplementary Figure S6

Supplementary Figure S7

Supplementary Table S1

Supplementary Table S2

Supplementary Table S3

Supplementary Figure Legends
