# Correction: LINC01016 promotes the malignant phenotype of endometrial cancer cells by regulating the miR-302a-3p/miR-3130-3p/NFYA/SATB1 axis

**DOI:** 10.1038/s41419-025-08385-3

**Published:** 2026-01-28

**Authors:** Xin Pan, Da Li, Jianing Huo, Fanfei Kong, Hui Yang, Xiaoxin Ma

**Affiliations:** Department of Obstetrics and Gynecology, Key Laboratory of Maternal-Fetal Medicine of Liaoning Province, Key Laboratory of Obstetrics and Gynecology of Higher Education of Liaoning Province, Shengjing Hospital of China Medical University, 39 Huaxiang Road, Shenyang, 110021 People’s Republic of China

Correction to: *Cell Death & Disease* 10.1038/s41419-018-0291-9, published online 21 February 2018

The two images, the fourth image in RL-95-2 group in Fig. 1 and the fifth image in RL-95-2 group in Fig. 2, were taken repeatedly from the same viewpoint and have duplicate parts. The original intent was to select a clearer and more aesthetically pleasing image to show in the paper, but an error occurred during the image insertion process. These corrections do not significantly impact the overall findings and conclusions of the paper.

Incorrect Fig. 1
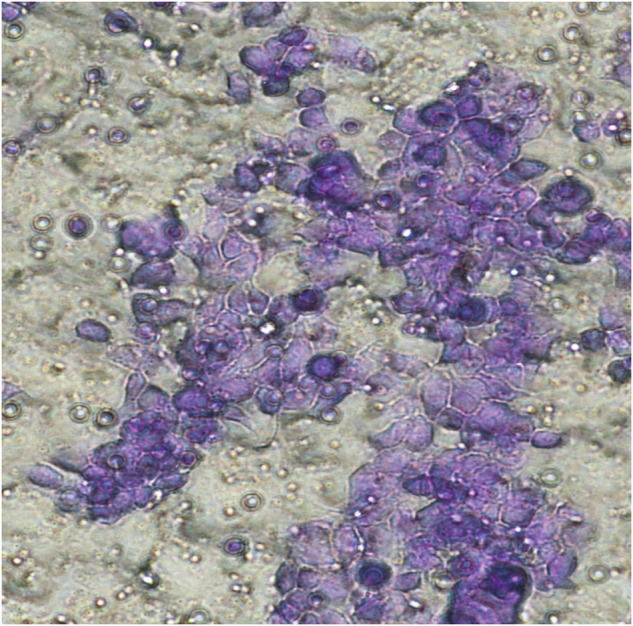


Correct Fig. 1
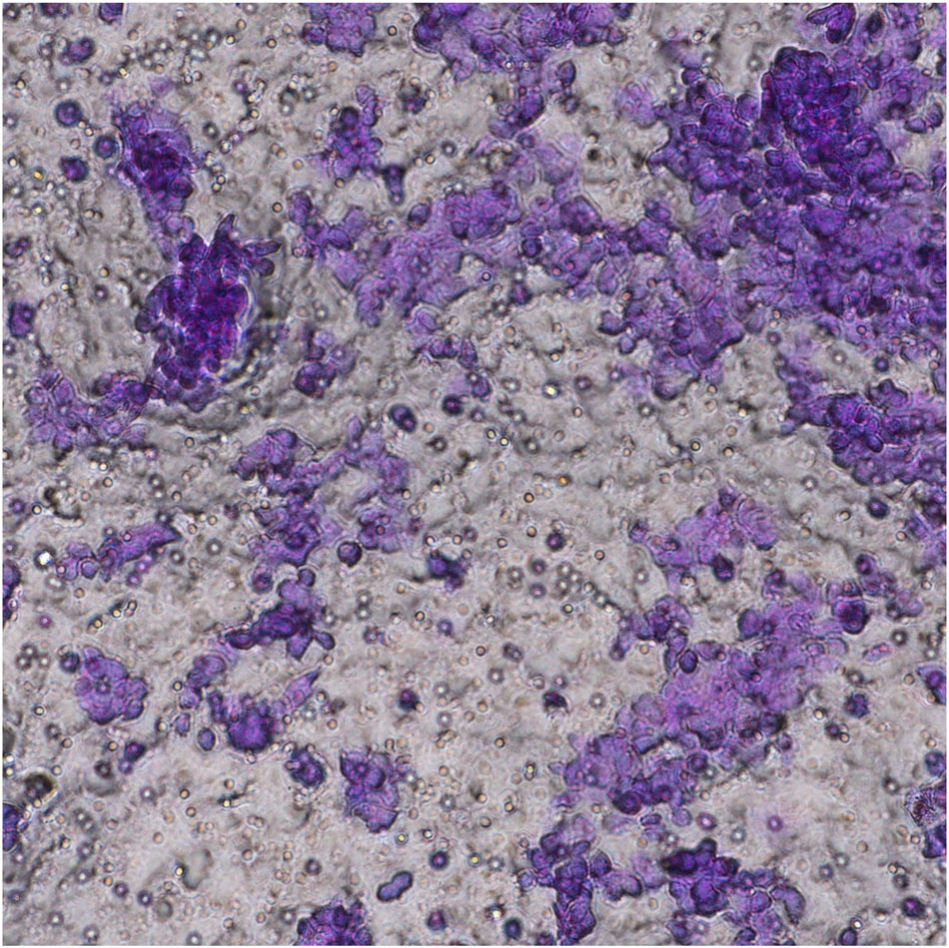


## Supplementary information

Original data

