# Supplementary figures and images for: LINC01016 promotes the malignant phenotype of endometrial cancer cells by regulating the miR-302a-3p/miR-3130-3p/NFYA/SATB1 axis
Source: Cell Death Dis. 2018 Feb 21;9(3):303. doi: 10.1038/s41419-018-0291-9 (PMC5833433; doi:10.1038/s41419-018-0291-9)

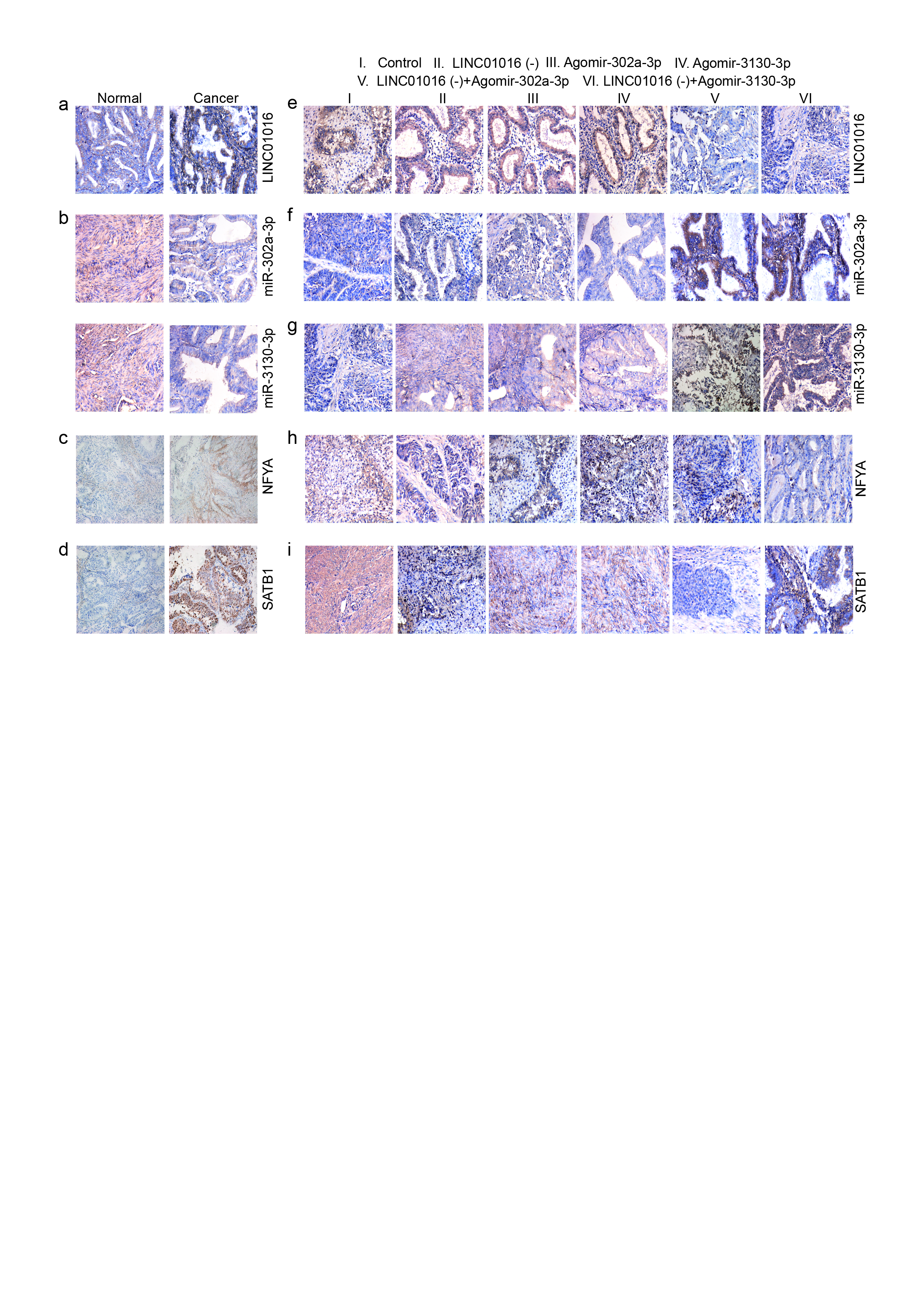

Supplement: Supplementary file 1 — Supplementary Figure S1 [file 41419_2018_291_MOESM1_ESM.tif]

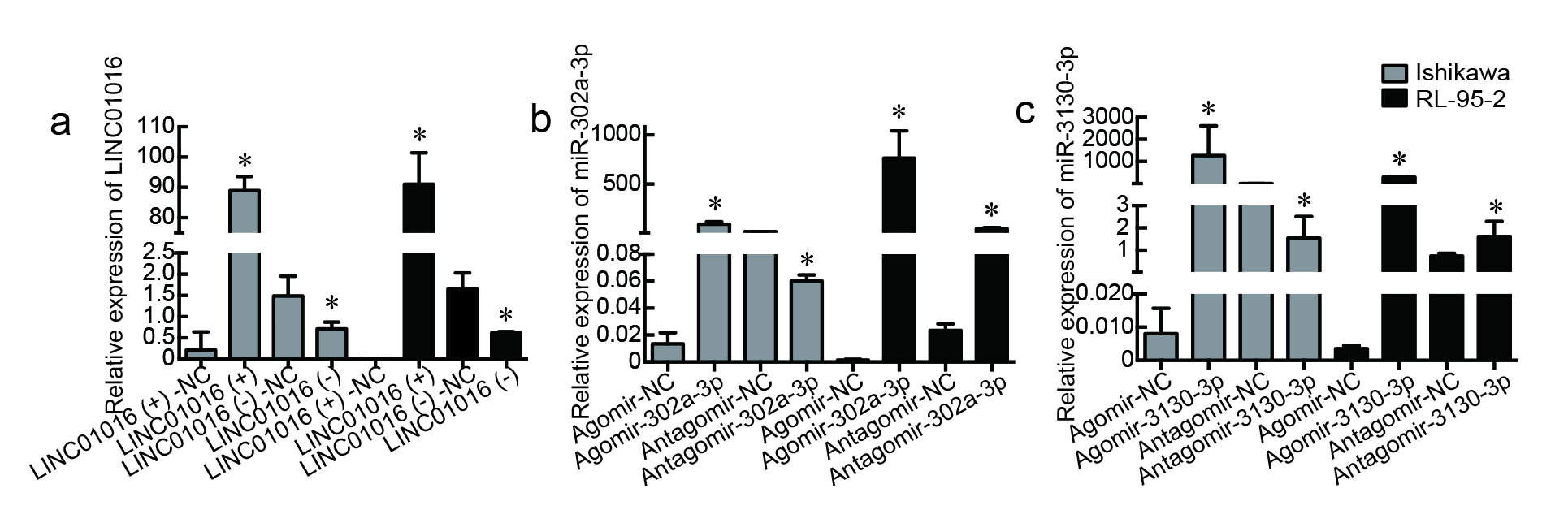

Supplement: Supplementary file 2 — Supplementary Figure S2 [file 41419_2018_291_MOESM2_ESM.tif]

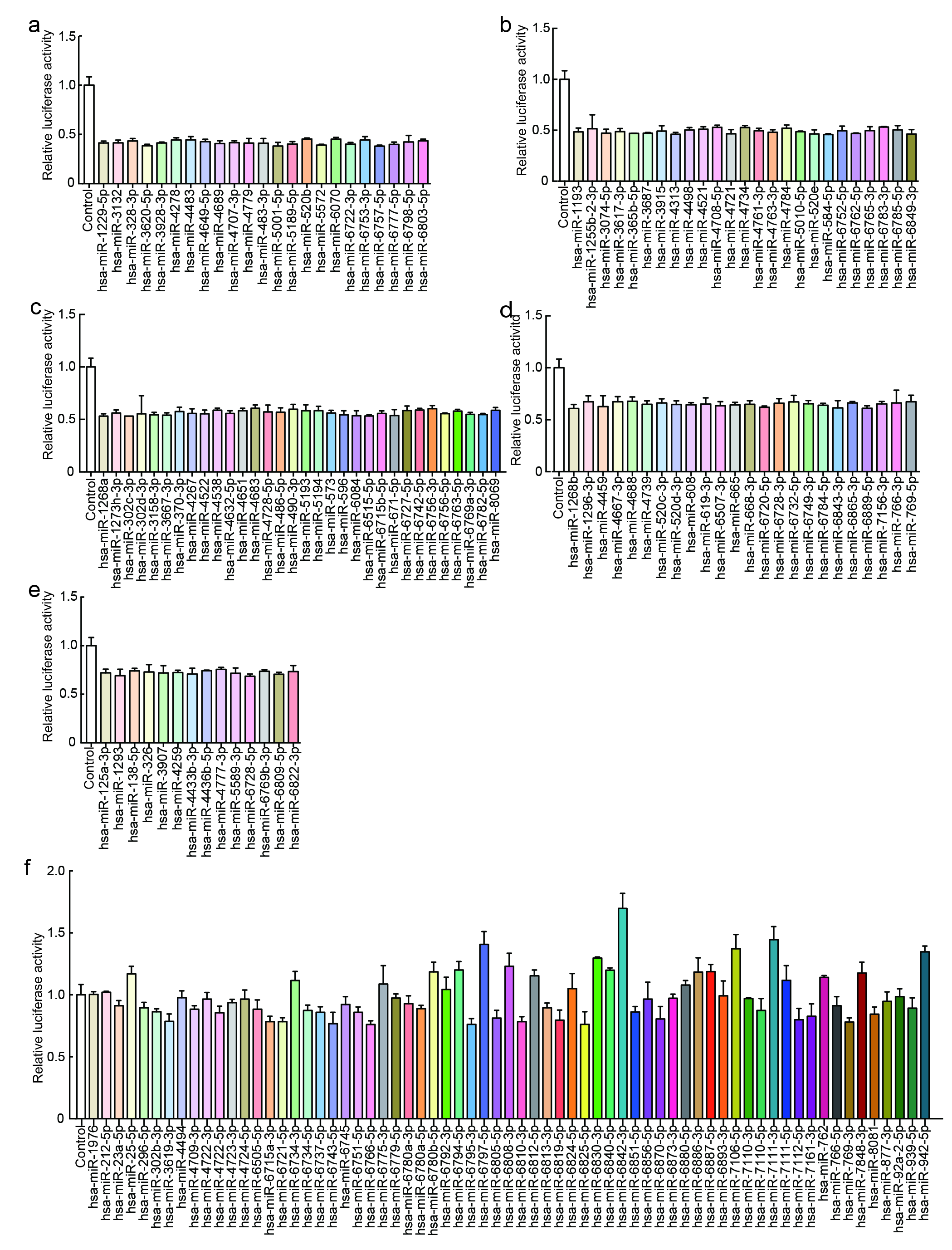

Supplement: Supplementary file 3 — Supplementary Figure S3 [file 41419_2018_291_MOESM3_ESM.tif]

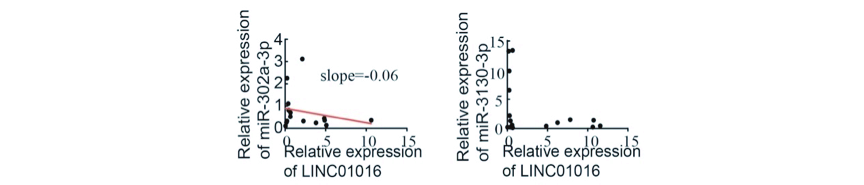

Supplement: Supplementary file 4 — Supplementary Figure S4 [file 41419_2018_291_MOESM4_ESM.tif]

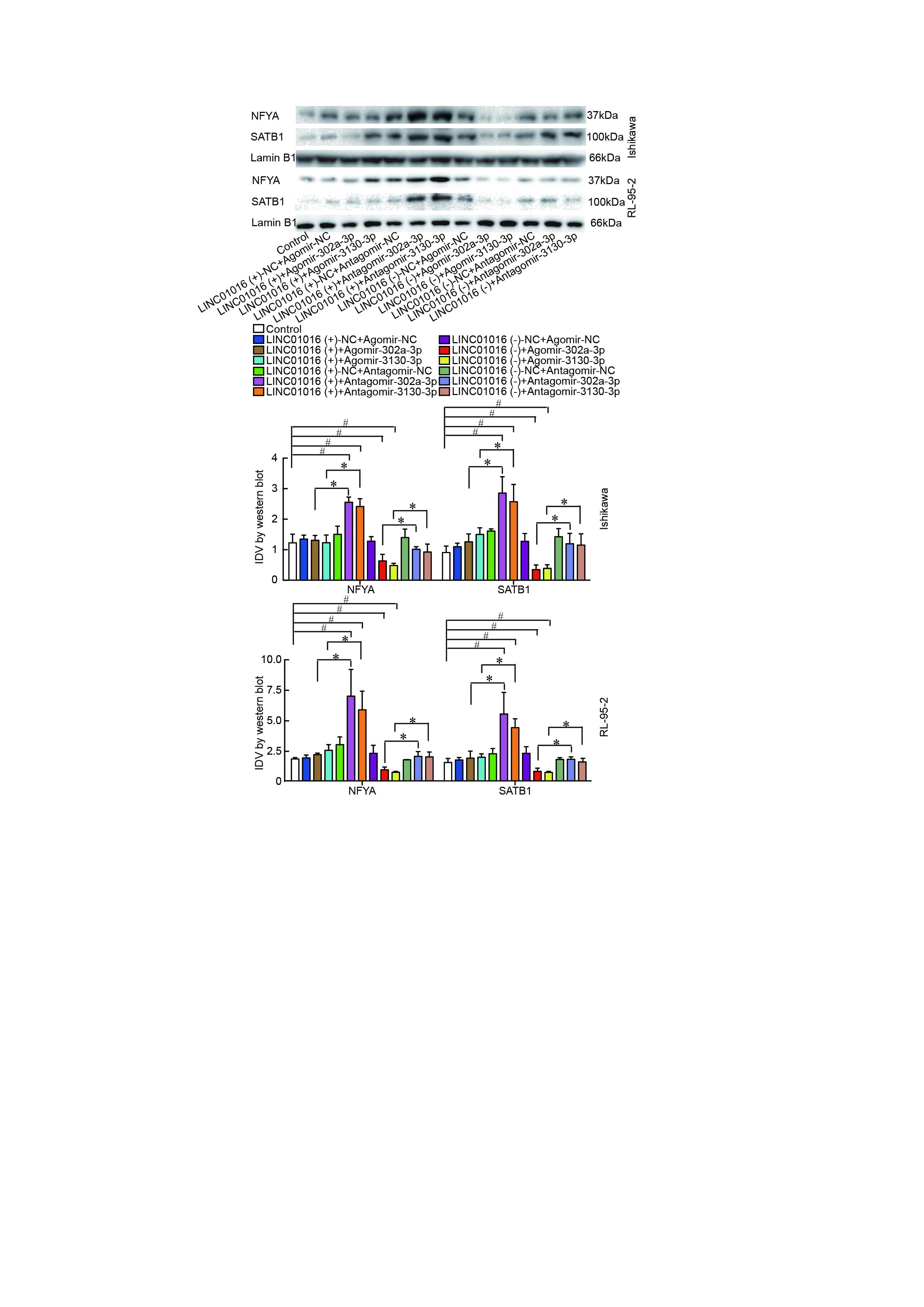

Supplement: Supplementary file 6 — Supplementary Figure S6 [file 41419_2018_291_MOESM6_ESM.tif]

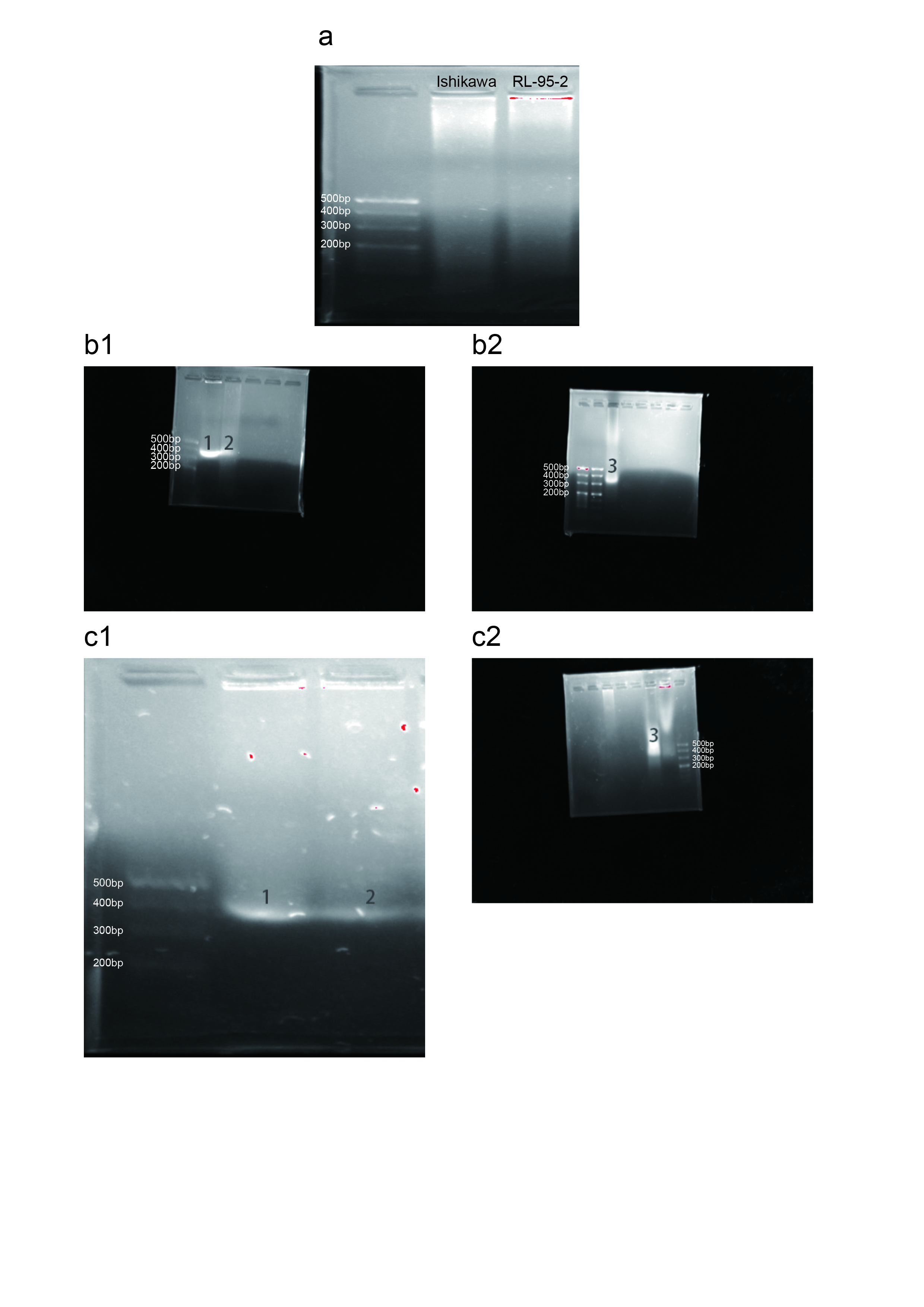

Supplement: Supplementary file 7 — Supplementary Figure S7 [file 41419_2018_291_MOESM7_ESM.tif]
