# Supplementary material for: LINC01016 promotes the malignant phenotype of endometrial cancer cells by regulating the miR-302a-3p/miR-3130-3p/NFYA/SATB1 axis
Source: Cell Death Dis. 2018 Feb 21;9(3):303. doi: 10.1038/s41419-018-0291-9 (PMC5833433; doi:10.1038/s41419-018-0291-9)
Supplement: Supplementary file 8 — Supplementary Table S1 [file 41419_2018_291_MOESM8_ESM.docx]

**Supplementary Table S1**

a. Relationship of LINC01016 expression with pathologic parameters of tumor.

| Clinical parameters | | low | high | *P* |
| --- | --- | --- | --- | --- |
| Age, y | <60 | 11 | 10 | .075 |
|  | ≥60 | 10 | 2 |  |
| Grade | I + II | 17 | 12 | .107 |
|  | III + IV | 4 | 0 |  |
| Differentiation | Low + Middle | 10 | 3 | .201 |
|  | High | 11 | 9 |  |
| Invasion depth | Superficial | 14 | 11 | .107 |
|  | Deep | 7 | 1 |  |
| Lymphatic metastasis | No | 19 | 11 | .909 |
|  | Yes | 2 | 1 |  |
| Vascular invasion | No | 18 | 12 | .258 |
|  | Yes | 2 | 0 |  |
| Distal metastasis | No | 20 | 11 | .679 |
|  | Yes | 1 | 1 |  |

b. Relationship of miR-302a-3p expression with pathologic parameters of tumor.

| Clinical parameters | | low | | high | *P* |
| --- | --- | --- | --- | --- | --- |
| Age, y | <60 | 19 | 2 | | .233 |
|  | ≥60 | 9 | 3 | |  |
| Grade | I + II | 24 | 4 | | .743 |
|  | III + IV | 4 | 1 | |  |
| Differentiation | Low + Middle | 14 | 2 | | .680 |
|  | High | 14 | 3 | |  |
| Invasion depth | Superficial | 22 | 5 | | .252 |
|  | Deep | 6 | 0 | |  |
| Lymphatic metastasis | No | 26 | 4 | | .357 |
|  | Yes | 2 | 1 | |  |
| Vascular invasion | No | 27 | 4 | | .156 |
|  | Yes | 1 | 1 | |  |
| Distal metastasis | No | 28 | 5 | | - |
|  | Yes | 0 | 0 | |  |

c. Relationship of hsa-miR-3130-3p expression with pathologic parameters of tumor.

| Clinical parameters | | low | | high | *P* |
| --- | --- | --- | --- | --- | --- |
| Age, y | <60 | 19 | 3 | | .186 |
|  | ≥60 | 7 | 4 | |  |
| Grade | I + II | 22 | 3 | | .042* |
|  | III + IV | 4 | 4 | |  |
| Differentiation | Low + Middle | 13 | 5 | | .413 |
|  | High | 13 | 2 | |  |
| Invasion depth | Superficial | 23 | 4 | | .093 |
|  | Deep | 3 | 3 | |  |
| Lymphatic metastasis | No | 24 | 5 | | .190 |
|  | Yes | 2 | 2 | |  |
| Vascular invasion | No | 24 | 6 | | .524 |
|  | Yes | 2 | 1 | |  |
| Distal metastasis | No | 25 | 7 | | 1.000 |
|  | Yes | 1 | 0 | |  |

**P* < 0.05.
