# Supplementary material for: LINC01016 promotes the malignant phenotype of endometrial cancer cells by regulating the miR-302a-3p/miR-3130-3p/NFYA/SATB1 axis
Source: Cell Death Dis. 2018 Feb 21;9(3):303. doi: 10.1038/s41419-018-0291-9 (PMC5833433; doi:10.1038/s41419-018-0291-9)
Supplement: Supplementary file 9 — Supplementary Table S2 [file 41419_2018_291_MOESM9_ESM.docx]

**Supplementary Table S2**

| Name | Sequence |
| --- | --- |
| sh-LINC01016-PSC50858-1 Sense | 5’-ccggTGGAACATTGGTTTAGTGTAActcgagTTACACTAAACCAATGTTC  CAtttttg-3’ |
| sh-LINC01016-PSC50858-1 Antisense | 5’-aattcaaaaaTGGAACATTGGTTTAGTGTAACTCGAGTTACACTAAACC  AATGTTCCA-3’ |
| sh-LINC01016-PSC50859-1 Sense | 5’-ccggAAGGGAATATACTAAGAAAGActcgagTCTTTCTTAGTATATTCCC  TTtttttg-3’ |
| sh-LINC01016-PSC50859-1 Antisense | 5’-aattcaaaaaAAGGGAATATACTAAGAAAGACTCGAGTCTTTCTTAGTAT  ATTCCCTT-3’ |
| sh-LINC01016-PSC50860-22 Sense | 5’-ccggGAGGAGATGAGCCGAGCAGTActcgagTACTGCTCGGCTCATCT  CCTCtttttg-3’ |
| sh-LINC01016-PSC50860-22 Antisense | 5’-aattcaaaaaGAGGAGATGAGCCGAGCAGTACTCGAGTACTGCTCGGC  TCATCTCCTC-3’ |
| miR-Stable Negative Control | Sense: 5’-UUCUCCGAACGUGUCACGUTT-3’ |
|  | Antisense: 5’-ACGUGACACGUUCGGAGAATT-3’ |
| Agomir-302a-3p | Sense: 5’-UAAGUGCUUCCAUGUUUUGGUGA-3’ |
|  | Antisense: 5’-ACCAAAACAUGGAAGCACUUAUU-3’ |
| Agomir-3130-3p | Sense: 5’-GCUGCACCGGAGACUGGGUAA-3’ |
|  | Antisense: 5’-ACCCAGUCUCCGGUGCAGCUU-3’ |
| miR-Inhibitor Negative Control | 5’-CAGUACUUUUGUGUAGUACAA-3’ |
| Antagomir-302a-3p | 5’-UCACCAAAACAUGGAAGCACUUA-3’ |
| Antagomir-3130-3p | 5’-UUACCCAGUCUCCGGUGCAGC-3’ |
| pGPU6/GFP/Neo-shNC | Sense: 5’-CACCGTTCTCCGAACGTGTCACGTCAAGAGATTACGTGACA  CGTTCGGAGAATTTTTTG-3’ |
|  | Antisense: 5’-GATCCAAAAAAGTTCTCCGAACGTGTCACGTAATCTCTTG  ACGTGACACGTTCGGAGAAC-3’ |
| pGPU6/GFP/Neo-NFYA-Homo-586 | Sense: 5’-CACCGCAGACCATCGTCTATCAACCTTCAAGAGAGGTTGATA  GACGATGGTCTGCTTTTTTG-3’ |
|  | Antisense: 5’-GATCCAAAAAAGCAGACCATCGTCTATCAACCTCTCTTGA  AGGTTGATAGACGATGGTCTGC-3’ |
| pGPU6/GFP/Neo-NFYA-Homo-685 | Sense: 5’-CACCGGCAGGAGCACAGATTGTTCATTCAAGAGATGAACAA  TCTGTGCTCCTGCCTTTTTTG-3’ |
|  | Antisense: 5’-GATCCAAAAAAGGCAGGAGCACAGATTGTTCATCTCTTG  AATGAACAATCTGTGCTCCTGCC-3’ |
| pGPU6/GFP/Neo-NFYA-Homo-769 | Sense: 5’-CACCGGCAGGCAATGTGGTCAATTCTTCAAGAGAGAATTGC  ACACATTGCCTGCCTTTTTTG-3’ |
|  | Antisense: 5’-GATCCAAAAAAGGCAGGCAATGTGGTCAATTCTCTCTTG  AAGAATTGACCACATTGCCTGCC-3’ |
| pGPU6/GFP/Neo-NFYA-Homo-896 | Sense: 5’-CACCGCCAAACAATACCACCGTATTTCAAGAGAATACGGTG  GTATTGTTTGGCTTTTTTG-3’ |
|  | Antisense: 5’-GATCCAAAAAAGCCAAACAATACCACCGTATTCTCTTGAA  ATACGGTGGTATTGTTTGGC-3’ |

Sequences of plasmid and RNA oligo/inhibitor.
