# Supplementary material for: LINC01016 promotes the malignant phenotype of endometrial cancer cells by regulating the miR-302a-3p/miR-3130-3p/NFYA/SATB1 axis
Source: Cell Death Dis. 2018 Feb 21;9(3):303. doi: 10.1038/s41419-018-0291-9 (PMC5833433; doi:10.1038/s41419-018-0291-9)
Supplement: Supplementary file 10 — Supplementary Table S3 [file 41419_2018_291_MOESM10_ESM.docx]

**Supplementary Table S3**

Primer sequence.

| Gene Name | Primer Sequence |
| --- | --- |
| LINC01016 | F: CAGGAGAGAGATGGGAGCAG  R: ATGGTTTAGGGGAGGAATGG |
| hsa-miR-302a-3p | F: AATAAGTGCTTCCATGTTTTGGTGA |
| hsa-miR-3130-3p | F: ATTGCTGCACCGGAGACTG |
| NFYA | F: GTGGTGAAGGTGGACGATTT  R: AGAAAGTTGGGTAGGACACTCG |
| SATB1 | F: TGCTCCCTCACTTTGTTTCC  R: GCCCAGTTGCCTACCAATAA |
| GAPDH | F: GCACCGTCAAGGCTGAGAAC  R: TGGTGAAGACGCCAGTGGA |
| U6 | F: CGGGTTTGTTTTGCATTTCT  R: AGTCCCAGCATGAACAGCTT |
| Primers of binding site 1 | F: TGGACACCTAAATACTGACTCTAAAAC  R: AAACTCCCCATTGGCTTTT |
| Primers of binding site 2 | F: AGGTCTGTCACATAACTCTTGGA  R: AGAAGGTAACAAACATCTAACTGCT |

F: Forward; R: Reverse.
