# Supplementary material for: LINC01016 promotes the malignant phenotype of endometrial cancer cells by regulating the miR-302a-3p/miR-3130-3p/NFYA/SATB1 axis
Source: Cell Death Dis. 2018 Feb 21;9(3):303. doi: 10.1038/s41419-018-0291-9 (PMC5833433; doi:10.1038/s41419-018-0291-9)
Supplement: Supplementary file 11 — Supplementary Figure Legends [file 41419_2018_291_MOESM11_ESM.docx]

**Supplementary Figure Legends**

**Supplementary Figure S1.** ISH for LINC01016 and miR-302a-3p/miR-3130-3p and IHC for NFYA and SATB1 in patients and xenograft tumour tissues.

**Supplementary Figure S2.** Verification of LINC01016 and miR-302a-3p/miR-3130-3p transfection by qRT-PCR.

**Supplementary Figure S3.** Different groups classified according to luciferase values compared to control. The value of the control was 1.0.

**Supplementary Figure S4.** Correlation between the expression levels of LINC01016 and miR-302a-3p/miR-3130-3p.

**Supplementary Table S1.** Relationship of LINC01016, miR-302a-3p and miR-3130-3p expression with pathologic tumour characteristics.

**Supplementary Figure S5.** LINC01016 and miR-302a-3p/miR-3130-3p mediate each other’s effects on malignant behavior.

**Supplementary Figure S6.** NFYA and SATB1 protein expression. **P* < 0.05 vs their corresponding negative control group; #*P* < 0.05 vs the control group without any treatment.

**Supplementary Figure S7.** The ultrasound results of two cell lines detected by DNA gel electrophoresis in two cell lines.

**Supplementary Table S2.** Sequences of the plasmids and RNA oligo/inhibitor used in cell transfection.

**Supplementary Table S3.** Primer sequences used for qRT-PCR.
