# Supplementary figures and images for: Correction: LINC01016 promotes the malignant phenotype of endometrial cancer cells by regulating the miR-302a-3p/miR-3130-3p/NFYA/SATB1 axis
Source: Cell Death Dis. 2026 Jan 28;17(1):146. doi: 10.1038/s41419-025-08385-3 (PMC12852104; doi:10.1038/s41419-025-08385-3)

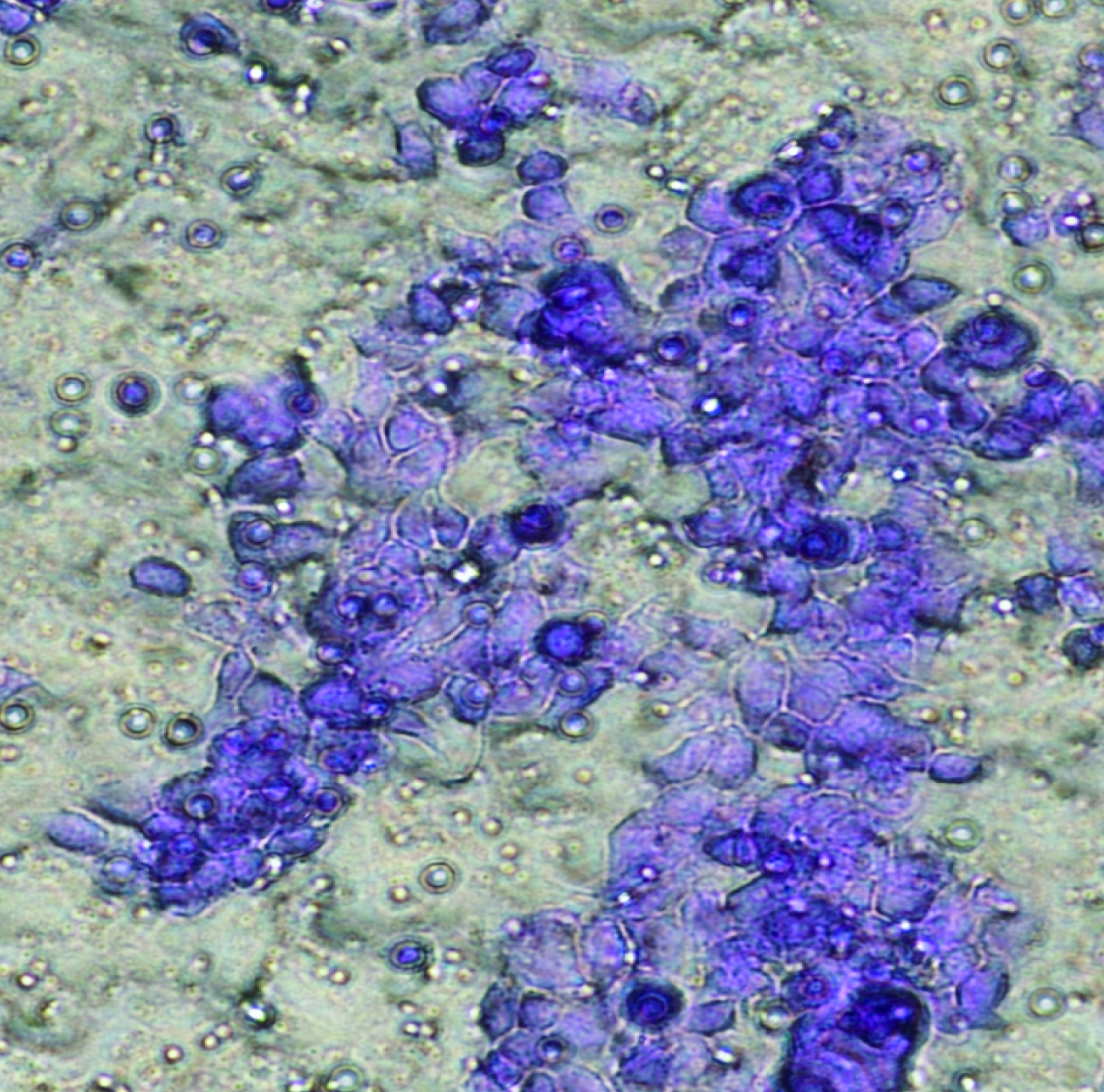

Supplement: Supplementary file 1 — Original data [file 41419_2025_8385_MOESM1_ESM.tif]
